# Dynamic Point Cloud Compression Based on Projections, Surface Reconstruction and Video Compression

**DOI:** 10.3390/s22010197

**Published:** 2021-12-28

**Authors:** Emil Dumic, Anamaria Bjelopera, Andreas Nüchter

**Affiliations:** 1 Department of Electrical Engineering, University North, 104. Brigade 3, 42000 Varaždin, Croatia; 2 Department of Electrical Engineering and Computing, University of Dubrovnik, Cira Carica 4, 20000 Dubrovnik, Croatia; anamaria.bjelopera@unidu.hr; 3 Department of Informatics VII—Robotics and Telematics, Julius-Maximilians-University Würzburg, 97074 Würzburg, Germany; andreas.nuechter@uni-wuerzburg.de

**Keywords:** 3DTK toolkit, map projections, point cloud compression, point-to-point measure, point-to-plane measure, Poisson surface reconstruction, octree

## Abstract

In this paper we will present a new dynamic point cloud compression based on different projection types and bit depth, combined with the surface reconstruction algorithm and video compression for obtained geometry and texture maps. Texture maps have been compressed after creating Voronoi diagrams. Used video compression is specific for geometry (FFV1) and texture (H.265/HEVC). Decompressed point clouds are reconstructed using a Poisson surface reconstruction algorithm. Comparison with the original point clouds was performed using point-to-point and point-to-plane measures. Comprehensive experiments show better performance for some projection maps: cylindrical, Miller and Mercator projections.

## 1. Introduction

A point cloud represents a set of discrete points in a given coordinate system, usually in a three-dimensional Cartesian system. It can represent different objects, urban settings and landscapes, or any other physical entities in different use cases such as: computer graphics and gaming, virtual reality, 3D content creation, medical applications, construction and manufacturing, consumer and retail, cultural heritage, remote sensing, autonomous vehicles, surveillance etc. [1]. Point clouds, together with light fields and digital holography are described as plenoptic representations of the visual scene, that are used in many immersive applications [2]. Point cloud processing algorithms can be considered in different scenarios, such as acquisition [3,4], coding and transmission [5,6] and (re)presentation and display [7,8].

Due to the data size of a static and dynamic point cloud, it is important to define efficient compression techniques for storage and compression. In this paper, we will present a dynamic point cloud compression that can be used in a point cloud transmission system. A basic overview has been presented in [9], while in [10] several static point clouds (with different sizes, e.g., number of points) have been compressed and decompressed using the equirectangular projection with a similar number of points as in the other used octree-based compression. In this paper we will present a comprehensive comparison of 10 different projection types, combined with the video compression algorithms for geometry and texture, and finally test surface reconstruction algorithms to fill the holes present after the lossy point cloud decompression process.

The structure of this article is as follows. Section 2 presents a related overview of point cloud compression algorithms. Section 3 presents different projection types that are used for projecting 3D point cloud to the image and vice versa. Section 4 describes the creation of the panorama image from a point cloud and how to recreate a point cloud from a panorama image. Section 5 gives the results from different projection types described in the previous section, using different objective quality measures and finally Section 6 gives the conclusions.

## 2. Related Work

Several static and dynamic point cloud coding solutions have been proposed recently. In [11] the authors describe an efficient octree implementation that is used to store, compress or visualize large point clouds. Storing is done without loss of precision, while reducing the size of a point cloud to half the original size. Also, 3D scan matching has been tested using the proposed method, by implementing the octree for nearest neighbor search (NNS) algorithm. The Random Sample Consensus (RANSAC) algorithm is also sped up by using the octree data structure. In the related work [12], shape registration algorithms have been compared using different NNS strategies, with the octree-based method being one of them. The Octree implementation (with arbitrarily chosen octree depth) is given in the “3DTK—The 3D Toolkit” [13].

Different from octree-based compression, in [14] the authors presented a point cloud compression algorithm based on projections. Different panorama generation methods have been tested, using several projection types: equirectangular, cylindrical, Mercator, rectilinear, Pannini, stereographic and Albers equal-area conic projections. It is shown that the reduced point clouds are useful for feature based registration on panorama images. In [15] point cloud compression scheme is proposed using panorama generated images and an equirectangular projection type. Range, color and reflectance information is encoded for each point, by using 24 bits for range (and storing it in an RGB image), 24 bits for color and 8 bits for reflectance. Also, lossless and lossy compression methods have been tested for obtained panorama images. In the case of lossy JPEG compression, an additional method is needed to remove artefacts from the decompressed point cloud.

Similarily, MPEG recently proposed two new point cloud compression codecs, named G-PCC (Geometry based Point Cloud Compression) codec [16] and V-PCC (Video based Point Cloud Compression) codec [17]. The G-PCC was created by merging two previously defined codecs, the L-PCC (LIDAR point cloud compression for dynamic point clouds) and the S-PCC (Surface point cloud compression for for static point clouds). Currently, G-PCC supports only intra prediction, so it does not use temporal redundancies. G-PCC compresses point clouds directly in 3D space. Currently, lossless mode provides up to a 10:1 compression ratio, while in lossy mode it is possible to obtain up to a 35:1 compression ratio with acceptable quality. In the MPEG’s V-PCC codec, the basic idea is to project point cloud from 3D to 2D, so that 2D projections are encoded using existing 2D video encoders such as H.265/HEVC video compression [18]. The current V-PCC encoder compresses dynamic point cloud with a compression ratio up to 125:1, with acceptable quality. More details about G-PCC and V-PCC are given in [19,20]. A comprehensive overview of G-PCC and V-PCC rate-distortion coding performance can be also found in [21]. A quality evaluation study of point cloud codecs G-PCC and V-PCC is presented in [22], showing the superior compression performance of the MPEG V-PCC compared to the MPEG G-PCC, for the selected static contents.

Neural network based point cloud compression schemes have also been proposed recently. In [23], the authors present point cloud compression using neural networks for separate and joint compression of geometry and texture. Better results are obtained for geometry and competitive results for color coding at low bitrates, comparing with CWI-PCL, the MPEG anchor codec is presented in [24]. In the paper [25], authors presented a new method for static point cloud geometric compression. It is based on the learned convolutional transform and uniform quantization. Compared to the MPEG reference software, the proposed algorithm achieves on average 51.5% BD-Rate (Bjöntegaard Delta Rate) savings, using the Microsoft Upper Body dataset [26]. The updated algorithm is presented in [27]. In the paper [28] a learned point cloud geometry compression is proposed, utilizing deep neural network-based variational autoencoders. The proposed algorithm shows higher compression efficiency than that of MPEG’s G-PCC, having at least 60% BD-Rate savings, tested on several datasets. The updated algorithm is proposed in [29]. Similar to [27] authors in [30] present the deep-learning coding approach to static point cloud geometry coding where a voxelized input point cloud is divided into 3D coding blocks of a fixed size and only non-empty 3D blocks are coded. The encoder transforms the input data into latent representations with lower dimensionality forcing a network to extract important features. The autoencoder learns the transform and its inverse operation suitable for the target data compared to image-transform coding where the transform basis functions are fixed. Performance results show improvements over the PCL (Point Cloud Library) [31]. The proposed compression from [30] is improved in [32] by adding the variational autoencoder which captures structure information still present on latent features and therefore entropy coding model parameters can be estimated on the encoder side and replicated on the decoder side more accurately. More importantly the authors add the resolution scalability via interlaced sub-sampling which not only increases the number of decoded points but also gives a good point cloud quality from a subjective point of view. Furthermore, these authors proposed adaptive deep learning-based static point cloud geometry coding which can adapt to any generic point cloud content to maximize the RD performance [33]. The author in [34] presents an adversarial autoencoding strategy for voxelized point cloud geometry where the main idea is to code each point cloud element independently and to decode it using a lower resolution reconstruction as side information. The encoder generates hash bytes, and the decoder combines them with side information to reconstruct the original block. The reconstructed block can be classified with an adversarial discriminator which is a regularizer for the reconstruction process thus improving the coding performance. A neural network architecture using a predictive coding module at the decoder stage for bit-rate reduction of geometry only point clouds is described in [35]. In this approach a block can be encoded independently or predicted using its neighbors based on the quality of the reconstructed block and the local topology of the model. Alternative to the mentioned block-by-block processing approaches point based models are used. In [36] the authors create an architecture consisting of a pointnet-based encoder, an uniform quantizer, an entropy estimation block and a nonlinear synthesis transformation module and in [37] a hierarhical autoencoder with multiscale loss function is presented. These architectures are insufficient for the processing of large point cloud data. VoxelDNN was proposed in [38] which combines the octree and voxel domains. Inference in this lossless compression is slow, and the occupancy probabilities are predicted sequentially, voxel by voxel, while the improved MSVoxelDNN models voxel occupancy and achieves rate savings over G-PCC up to 17% on average [39]. One of the new methods is presented in [40] and it brings a solution that can be applied to both static and dynamic point cloud compression. It employs the voxel context based entropy model and for dynamic PC compression temporal dependency is exploited. In the work of [41] detachable learning-based residual coding solution is created where the residual module enhances the decoded model quality at the expense of added bitrate.

## 3. Projection Types and Their Description

In this section, we describe different projection types and their inverse solutions that is later used in the process of obtaining 2D image projection from point clouds. Several of them are adopted from [15,42,43]. For each projection type, example panorama images for the geometry and texture are created using the point cloud “longdress_vox10_1060.ply” from *Longdress* dynamic point cloud dataset [44].

A point cloud geometry is first transformed from a Cartesian to a spherical coordinate system. Afterwards, 2D geometry panorama image coordinates (row number *x* and column number *y*) are calculated from the angle information (longitude θ and latitude φ), while the intensity (bit depth) of the 2D geometry panorama represents radius in spherical coordinates. An attribute panorama image, i.e., a 2D image representing color information in our case, is created by using the same row number *x* and column number *y* as for the geometry, with the same color as the point cloud point that it represents.

### 3.1. Lambert Azimuthal Equal-Area Projection

Transformation equations and the inverse formulas are given in Equations (1) and (2). $\varphi_{1}$ is the standard parallel, while $\theta_{0}$ is the central longitude.

(1) $$\begin{array}{cc} \; & x=k^{\prime }\cos\varphi \sin\left(\theta -\theta_{0}\right),\\ \; & y=k^{\prime }\left(\cos\varphi_{1}\sin\varphi -\sin\varphi_{1}\cos\varphi \cos\left(\theta -\theta_{0}\right)\right),\\ \; & k^{\prime }=\sqrt{\frac{2}{1+\sin\varphi_{1}\sin\varphi +\cos\varphi_{1}\cos\varphi \cos\left(\theta -\theta_{0}\right)}}. \end{array}$$

The inverse formulas are given in Equation (2):

(2) $$\begin{array}{cc} \; & \theta =\arctan\frac{x\sin C}{\rho \cos\varphi_{1}\cos C-y\sin\varphi_{1}\sin C}+\theta_{0},\\ \; & \varphi =\arcsin\left(\cos C\sin\varphi_{1}+\frac{y\sin C\cos\varphi_{1}}{\rho }\right),\\ \; & \rho =\sqrt{x^{2}+y^{2}},\\ \; & C=2\arcsin\frac{\rho }{2}. \end{array}$$

**Figure 1:**
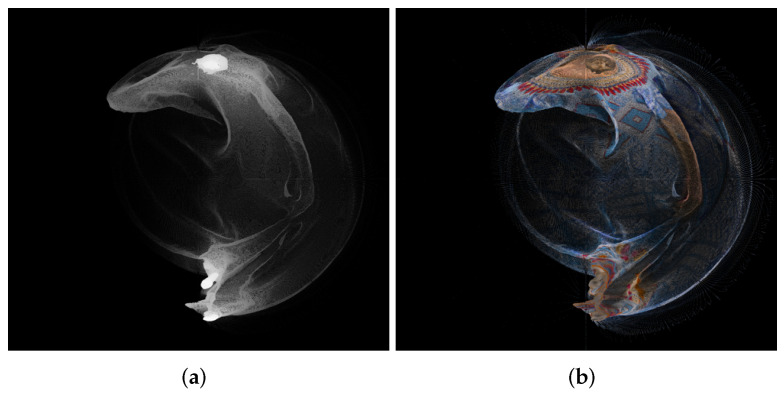
Panorama images created using point cloud “longdress_vox10_1060.ply” from the Lambert azimuthal equal-area projection, for the geometry (**a**) and texture (**b**).

An example of panorama images is given in Figure 1.

### 3.2. Albers Equal-Area Conic Projection

Transformation equations and the inverse formulas are given in Equations (3) and (4). Usually, $\left(\varphi_{0},\theta_{0}\right)=\left(0^{\circ },0^{\circ }\right)$, while $\varphi_{1}$ and $\varphi_{2}$ represent minimum and maximum latitude.

$$\begin{array}{cc} \; & x=\rho \sin\left(N\left(\theta -\theta_{0}\right)\right),\\ \; & y=\rho_{0}-\rho \cos\left(N\left(\theta -\theta_{0}\right)\right), \end{array}$$

where:

(3) $$\begin{array}{cc} \; & N=\frac{1}{2}\left(\sin\varphi_{1}+\sin\varphi_{2}\right),\\ \; & C=\cos{}^{2}\varphi_{1}+2N\sin\varphi_{1},\\ \; & \rho_{0}=\frac{1}{N}\sqrt{C-2N\sin\varphi_{0}},\\ \; & \rho =\frac{1}{N}\sqrt{C-2N\sin\varphi }. \end{array}$$

The inverse formulas are given in Equation (4):

(4) $$\begin{array}{cc} \; & \theta =\theta_{0}+\frac{1}{N}\arctan\frac{x}{\rho_{0}-y},\\ \; & \varphi =\arcsin\frac{C-\left(x^{2}+{\left(\rho_{0}-y\right)}^{2}\right)N^{2}}{2N}. \end{array}$$

**Figure 2:**
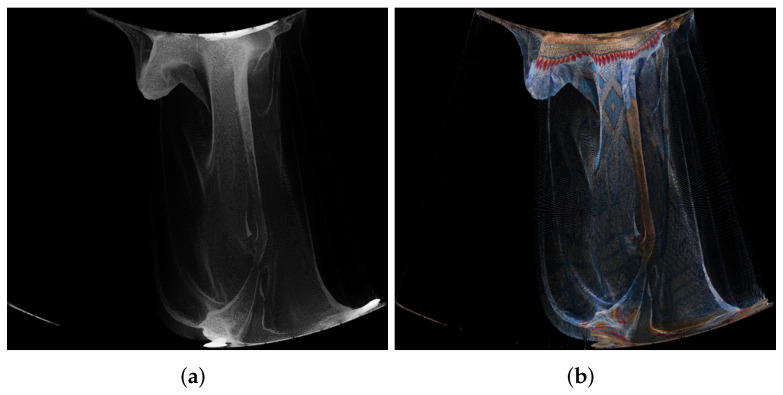
Panorama images created using point cloud “longdress_vox10_1060.ply” from the Albers equal-area conic projection, for the geometry (**a**) and texture (**b**).

An example of panorama images is given in Figure 2.

### 3.3. Cylindrical Projection

Cylindrical projection is similar to the equirectangular projection, however vertical coordinate is tangent of the latitude. Transformation equations and the inverse formulas are given in Equations (5) and (6). Usually, $\left(\varphi_{0},\theta_{0}\right)=\left(0^{\circ },0^{\circ }\right)$. Later in the experiments, we will “compress” all φ latitude angles before creating panorama images, i.e., multiply φ with 0.825, and “decompress” (divide by 0.825) after recreating point cloud. This is because tangent function is not defined for angles near $\pm 90^{\circ }$.

(5) $$\begin{array}{cc} \; & x=\theta -\theta_{0},\\ \; & y=\tan\varphi -\tan\varphi_{0}. \end{array}$$

The inverse formulas are given in Equation (6):

(6) $$\begin{array}{cc} \; & \theta =x+\theta_{0},\\ \; & \phi =\arctan(y+\tan\varphi_{0}). \end{array}$$

**Figure 3:**
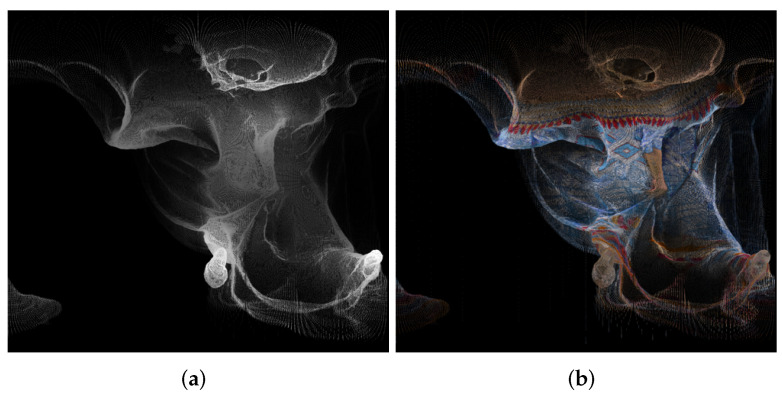
Panorama images created using point cloud “longdress_vox10_1060.ply” from the cylindrical projection, for the geometry (**a**) and texture (**b**).

An example of panorama images is given in Figure 3.

### 3.4. Cylindrical Equal-Area Projection

Transformation equations and the inverse formulas are given in Equations (7) and (8). $\theta_{0}$ is the standard longitude, while $\varphi_{s}$ is the standard latitude, e.g., for $\varphi_{s}$ = 0 it is called Lambert cylindrical equal-area projection.

(7) $$\begin{array}{cc} \; & x=\left(\theta -\theta_{0}\right)cos\varphi_{s},\\ \; & y=\frac{\sin\varphi }{\cos\varphi_{s}}. \end{array}$$

The inverse formulas are given in Equation (8):

(8) $$\begin{array}{cc} \; & \theta =\frac{x}{\cos\varphi_{s}}+\theta_{0},\\ \; & \varphi =\arcsin(y\cos\varphi_{s}). \end{array}$$

**Figure 4:**
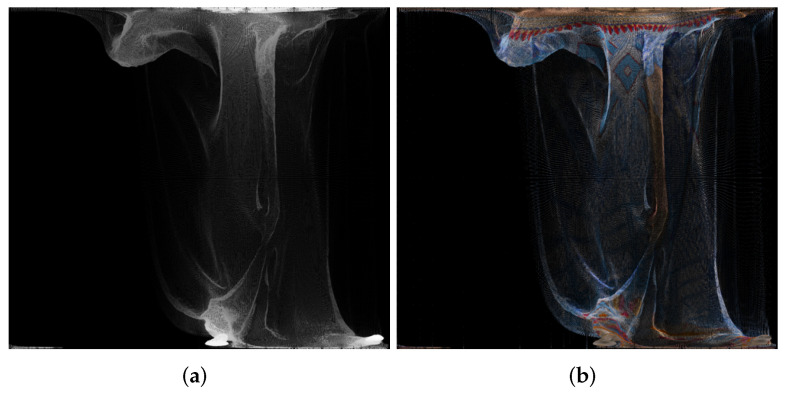
Panorama images created using point cloud “longdress_vox10_1060.ply” from the cylindrical equal-area projection, for the geometry (**a**) and texture (**b**).

An example of panorama images is given in Figure 4.

### 3.5. Equidistant Cylindrical Projection

Transformation equations and the inverse formulas for equidistant cylindrical projection are given in Equations (9) and (10). An equirectangular projection, one of the most common projection types, is a type of cylindrical equidistant projection. Horizontal coordinate *x* in this projection type is the longitude θ, while the vertical coordinate *y* is the latitude φ. $\varphi_{1}$ represents standard parallels (north and south of the equator) where the scale of the projection is true. For $\left(\varphi_{0},\theta_{0}\right)=\left(0^{\circ },0^{\circ }\right)$ and $\cos\varphi_{1}$ = 0 it is called the equirectangular projection, Equation (9).

(9) $$\begin{array}{cc} \; & x=\left(\theta -\theta_{0}\right)\cos\varphi_{1},\\ \; & y=\varphi -\varphi_{0}. \end{array}$$

The inverse formulas are given in Equation (10):

(10) $$\begin{array}{cc} \; & \theta =\frac{x}{\cos\varphi_{1}}+\theta_{0},\\ \; & \phi =y+\phi_{0}. \end{array}$$

**Figure 5:**
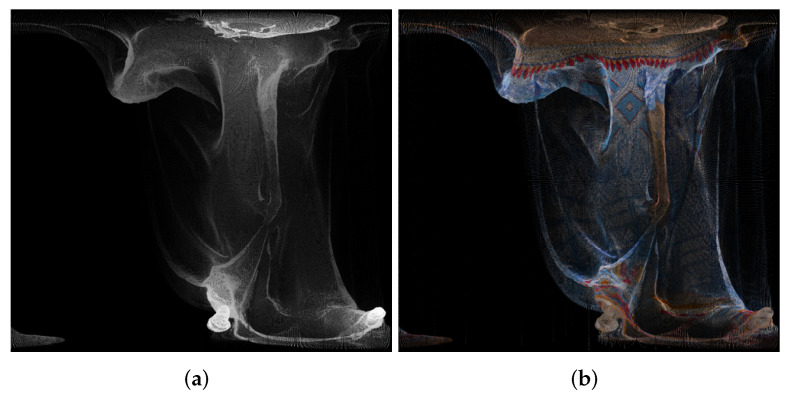
Panorama images created using point cloud “longdress_vox10_1060.ply” from the equirectangular projection, for the geometry (**a**) and texture (**b**).

An example of panorama images is given in Figure 5.

### 3.6. Mercator Projection

Transformation equations and the inverse formulas are given in Equations (11) and (12). Problems may arise at latitudes φ near $\pm 90^{\circ }$. Usually, $\left(\varphi_{0},\theta_{0}\right)=\left(0^{\circ },0^{\circ }\right)$. Similar to the cylindrical projection, later in the experiments we will “compress” all φ latitude angles, multiplying them with the factor 0.825, to create a panorama image (and “decompress”—divide them with the factor 0.825 to recreate point clouds).

(11) $$\begin{array}{cc} \; & x=\theta -\theta_{0},\\ \; & y=\ln\left(\tan\varphi +\frac{1}{\cos\varphi }\right)-y_{0},\\ \; & y_{0}=\ln\left(\tan\varphi_{0}+\frac{1}{\cos\varphi_{0}}\right) \end{array}$$

The inverse formulas are given in Equation (12):

(12) $$\begin{array}{cc} \; & \theta =x+\theta_{0},\\ \; & \varphi =2\arctan e^{y+y_{0}}-\frac{\pi }{2}. \end{array}$$

**Figure 6:**
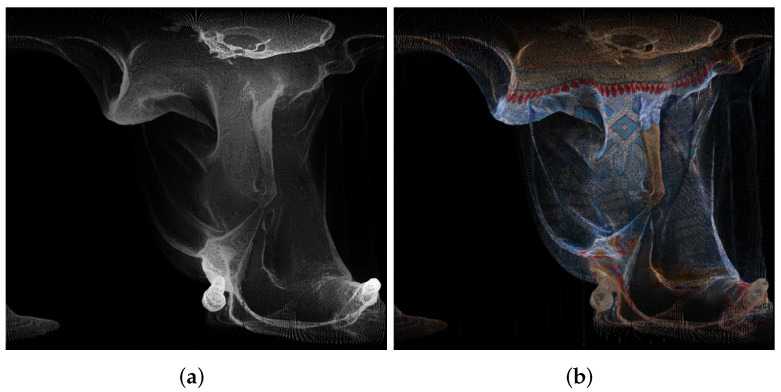
Panorama images created using point cloud “longdress_vox10_1060.ply” from the Mercator projection, for the geometry (**a**) and texture (**b**).

An example of panorama images is given in Figure 6.

### 3.7. Miller Projection

The Miller projection is a modified Mercator projection. Transformation equations and the inverse formulas are given in Equations (13) and (14). Problems that may arise using the Mercator projection for latitudes ϕ near $\pm 90^{\circ }$ are not present in the Miller projection. Usually, $\left(\phi_{0},\theta_{0}\right)=\left(0^{\circ },0^{\circ }\right)$.

(13) $$\begin{array}{cc} \; & x=\theta -\theta_{0},\\ \; & y=\frac{5}{4}\ln\left[\tan\left(\frac{\pi }{4}+\frac{2\varphi }{5}\right)\right]-y_{0}.\\ \; & y_{0}=\frac{5}{4}\ln\left[\tan\left(\frac{\pi }{4}+\frac{2\varphi_{0}}{5}\right)\right]. \end{array}$$

The inverse formulas are given in Equation (14):

(14) $$\begin{array}{cc} \; & \theta =x+\theta_{0},\\ \; & \varphi =\frac{5}{2}\arctan e^{\frac{4(y+y_{0})}{5}}-\frac{5\pi }{8}. \end{array}$$

**Figure 7:**
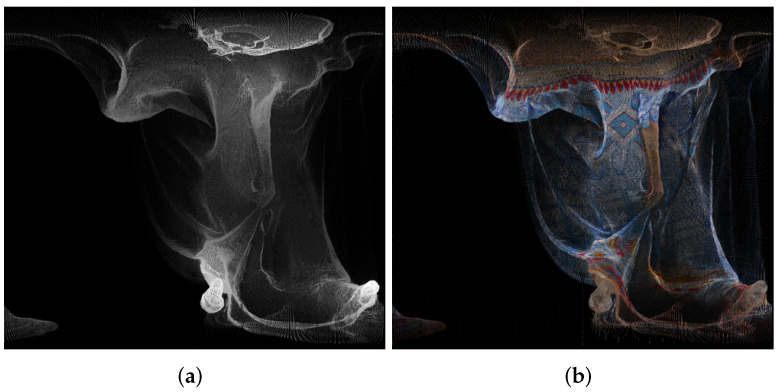
Panorama images created using point cloud “longdress_vox10_1060.ply” from the Miller projection, for the geometry (**a**) and texture (**b**).

An example of panorama images is given in Figure 7.

### 3.8. Rectilinear Projection

Transformation equations and the inverse formulas are given in Equations (15) and (16). $\theta_{0}$ and $\varphi_{1}$ are the longitude and latitude of the center of the projection. It is recommended to use this projection for the horizontal and vertical angles of less than $120^{\circ }$. Therefore, the panorama has to be divided into smaller subsets, e.g., minimum 3 (later we will use 4). In addition, for greater vertical angles, all angles are scaled to fit in less than $\pm 90^{\circ }$. Later in the experiments, similar to the cylindrical and Mercator projections, we will “compress” and “decompress” all of the φ latitude angles, by multiplying and dividing them by 0.825.

(15) $$\begin{array}{cc} \; & x=\frac{\cos\varphi \sin\left(\theta -\theta_{0}\right)}{\sin\varphi_{1}\sin\varphi +\cos\varphi_{1}\cos\varphi \cos\left(\theta -\theta_{0}\right)},\\ \; & y=\frac{\cos\varphi_{1}\sin\varphi -\sin\varphi_{1}\cos\varphi \cos\left(\theta -\theta_{0}\right)}{\sin\varphi_{1}\sin\varphi +\cos\varphi_{1}\cos\varphi \cos\left(\theta -\theta_{0}\right)}. \end{array}$$

The inverse formulas are given in Equation (16):

(16) $$\begin{array}{cc} \; & \theta =\theta_{0}+\arctan\frac{x\sin C}{\rho \cos\varphi_{1}\cos C-y\sin\varphi_{1}\sin C},\\ \; & \varphi =\arcsin\left(\cos C\sin\varphi_{1}+\frac{y\sin C\cos\varphi_{1}}{\rho }\right),\\ \; & \rho =\sqrt{x^{2}+y^{2}},\\ \; & C=\arctan\rho . \end{array}$$

**Figure 8:**
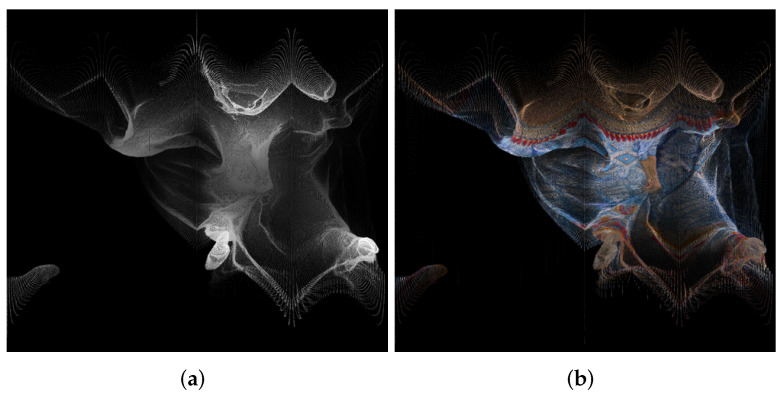
Panorama images created using point cloud “longdress_vox10_1060.ply” from the rectilinear projection, for the geometry (**a**) and texture (**b**).

An example of panorama images is given in Figure 8.

### 3.9. Pannini Projection

Transformation equations and the inverse formulas are given in Equations (17) and (18). $\theta_{0}$ and $\varphi_{1}$ are the longitude and latitude of the center of the projection. Parameter *d* can be any non-negative number. For d=0 we have a rectilinear projection, while d=1 is the usual Pannini projection, used in the later experiments. It is recommended to use this projection for horizontal and vertical angles of less than $150^{\circ }$. Therefore, the panorama has to be divided into smaller subsets, e.g., minimum of 3 (later we will use 4). In addition, for greater vertical angles, all angles are scaled to fit in less than $\pm 90^{\circ }$. For this projection type, later in the experiments we will “compress” and “decompress” all φ latitude angles, multiplying and dividing them with the factor 0.825.

(17) $$\begin{array}{cc} \; & x=\frac{\left(d+1\right)\sin\left(\theta -\theta_{0}\right)}{d+\sin\varphi_{1}\tan\varphi +\cos\varphi_{1}\cos\left(\theta -\theta_{0}\right)},\\ \; & y=\frac{\left(d+1\right)\tan\varphi (\cos\varphi_{1}-\sin\varphi_{1}\left(\frac{1}{\tan\varphi }\right)\cos(\theta -\theta_{0}))}{d+\sin\varphi_{1}\tan\varphi +\cos\varphi_{1}\cos\left(\theta -\theta_{0}\right)}. \end{array}$$

The inverse formulas are given in Equation (18):

(18) $$\begin{array}{cc} \; & A=\frac{y}{x\cos\varphi_{1}},\\ \; & B=\tan\varphi_{1},\\ \; & C=Ax\sin\varphi_{1}-d-1,\\ \; & D=Bx\sin\varphi_{1}+x\cos\varphi_{1},\\ \; & E=-xd. \end{array}$$

Finally we yield, Equation (19):

(19) $$\begin{array}{cc} \; & C\sin\left(\theta -\theta_{0}\right)+D\cos\left(\theta -\theta_{0}\right)=E\\ \; & \theta =\theta_{0}+\arccos\left(\frac{E}{\sqrt{C^{2}+D^{2}}}\right)+\arctan\left(\frac{C}{D}\right)\\ \; & \varphi =\arctan\left(A\sin\left(\theta -\theta_{0}\right)+B\cos\left(\theta -\theta_{0}\right)\right). \end{array}$$

**Figure 9:**
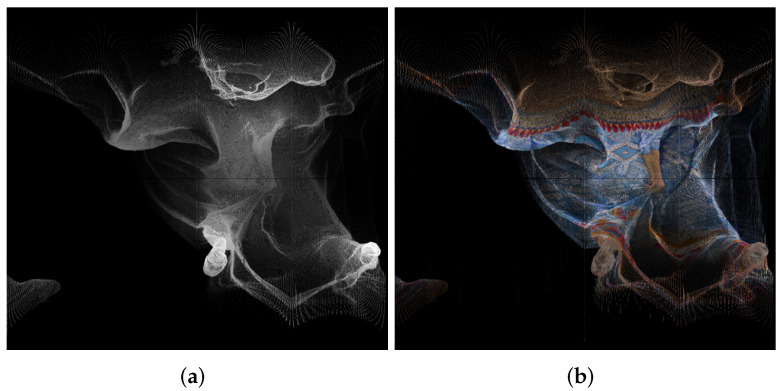
Panorama images created using point cloud “longdress_vox10_1060.ply” from the Pannini projection, for the geometry (**a**) and texture (**b**).

An example of panorama images is given in Figure 9.

### 3.10. Stereographic Projection

Transformation equations and the inverse formulas are given in Equations (20) and (21). $\theta_{0}$ and $\varphi_{1}$ are the longitude and latitude of the center of the projection. It is advisable to use a $120^{\circ }$ longitude, e.g., to divide the image into at least 3 subsets (later we will use 4).

(20) $$\begin{array}{cc} \; & x=\frac{2R\cos\varphi \sin\left(\theta -\theta_{0}\right)}{1+\sin\varphi_{1}\sin\varphi +\cos\varphi_{1}\cos\varphi \cos\left(\theta -\theta_{0}\right)},\\ \; & y=\frac{2R(\cos\varphi_{1}\sin\varphi -\sin\varphi_{1}\cos\varphi \cos\left(\theta -\theta_{0}\right))}{1+\sin\varphi_{1}\sin\varphi +\cos\varphi_{1}\cos\varphi \cos\left(\theta -\theta_{0}\right)}. \end{array}$$

The inverse formulas are given in Equation (21):

(21) $$\begin{array}{cc} \; & \theta =\theta_{0}+\arctan\frac{x\{inC}{\rho \cos\varphi_{1}\cos C-y\sin\varphi_{1}\sin C},\\ \; & \varphi =\arcsin\left(\cos C\sin\varphi_{1}+\frac{y\sin C\cos\varphi_{1}}{\rho }\right),\\ \; & \rho =\sqrt{x^{2}+y^{2}},\\ \; & C=2\arctan\left(\frac{\rho }{2R}\right). \end{array}$$

**Figure 10:**
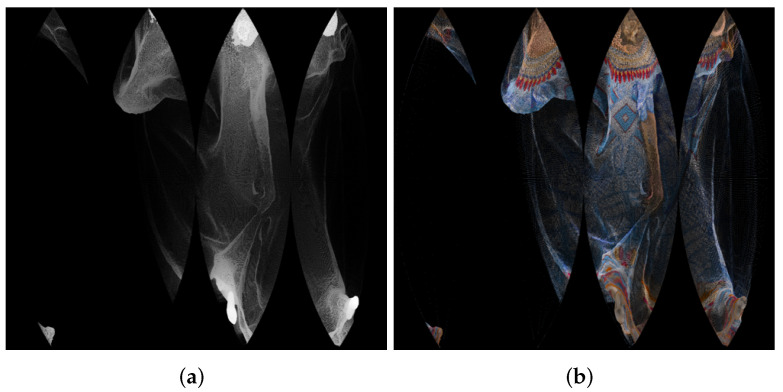
Panorama images created using point cloud “longdress_vox10_1060.ply” from the stereographic projection, for the geometry (**a**) and texture (**b**).

An example of panorama images is given in Figure 10.

## 4. Creation of Panorama Images from Point Clouds and Recreation of Point Clouds

In this section we will describe how to calculate panorama images from point clouds, as well as how to recreate point clouds from obtained panorama images. Programs that were used are 3DTK toolkit [13], MeshLab [45], CloudCompare [46], FFmpeg [47] and MATLAB for scripts.

For this example, we will use the Miller projection, although any other projection described earlier can be used in a similar way. Also, we will use *Longdress* point cloud, first 20 frames [44]. This point cloud is a voxelized point cloud with a bounding box size of 1024 × 1024 × 1024, i.e., with 10-bit precision per each coordinate. Additionally, texture (color) for each point is represented with 24-bit RGB format, i.e., with 8 bits per color channel. Overall, 3×10+3×8=54 bits per point are used, in the ideal case; however, depending on the used format, even the binary format might occupy much more space, as we present later.

The input point cloud is read in MATLAB and an offset is added to all its points, so that the bounding box center is at (0, 0, 0). Afterwards, it is scaled so that the maximum distance between any point and the origin (0, 0, 0) does not exceed $2^{bit\_num}-1=65,535$ for a 16 bit panorama grayscale image, Equation (22).

(22) $$\begin{array}{cc} \; & \mathrm{scale}\_\mathrm{factor}=\frac{K\cdot (2^{\mathrm{bit}\_\mathrm{num}}-1)}{\max\left(\sqrt{\left(xx{\left(i\right)}^{2}+yy{\left(i\right)}^{2}+zz{\left(i\right)}^{2}\right)}\right)}\\ \; & \mathrm{bit}\_\mathrm{num}=16\;(\mathrm{in}\;\mathrm{the}\;\mathrm{later}\;\mathrm{case},\;\mathrm{but}\;\mathrm{could}\;\mathrm{be}\;\mathrm{also}\;24\;\mathrm{for}\;\mathrm{some}\;\mathrm{other}\;\mathrm{point}\;\mathrm{clouds})\\ \; & K=0.01\;(\mathrm{due}\;\mathrm{to}\;\mathrm{the}\;\mathrm{later}\;\mathrm{scaling}\;\mathrm{made}\;\mathrm{by}\;\mathrm{the}\;3\mathrm{DTK}\;\mathrm{toolkit})\\ \; & i\in \{1,\ldots ,N\},N=\;\mathrm{number}\;\mathrm{of}\;\mathrm{points}\\ \; & xx(i),yy(i),zz(i)\;\mathrm{are}\;\mathrm{Cartesian}\;\mathrm{coordinates}\;\mathrm{of}\;\mathrm{the}\;\mathrm{point}\;i \end{array}$$

Both geometry and texture are represented using the same projection. In the case of geometry, we use 16 bit input grayscale .png for the later compression. However, approximately 9 bits may be enough for the tested point cloud: the maximum distance from the center is 512, before scaling, but the number may not be integer, so some precision loss may occur for the 9 bit representation. For larger point clouds, 24-bit representation may also be used, which is also implemented in the 3DTK toolkit, storing geometry in an RGB image [15]. In the case of texture, we additionally create a Voronoi diagram using OpenCV “distanceTransform” function and L2 norm (while creating the texture image in the 3DTK toolkit), Listing 1. A Voronoi diagram (tessellation, decomposition) is the partitioning of a plane with n points into convex polygons with exactly one generating point in each polygon and every point in a particular polygon being closer to its generating point than any other generating point [48]. By creating a “full” texture image, instead of originally sparse, additional video compression efficiency may be achieved later.

**Listing 1:** Voronoi diagram creation.


*//create geometry and texture image using 3DTK* *//get mask as the inverse of the existing pixels from~geometry*

distanceTransform(mask, distance, labels, CV_DIST_L2, CV_DIST_MASK_PRECISE, CV_DIST_LABEL_PIXEL);

*//map labels to indices* std::vector<cv::Vec2i> label_to_index;

*//reserve memory for faster push_back* label_to_index.reserve(sum(~mask)[0]);

**for** (**int** row = 0; row < mask.rows; ++row) **for** (**int** col = 0; col < mask.cols; ++col) **if** (mask.at<uchar>(row, col) == 0) *//this pixel exist* label_to_index.push_back(cv::Vec2i(row, col));

*//create ``full’’ image* **for** (**int** row = 0; row < mask.rows; ++row) { **for** (**int** col = 0; col < mask.cols; ++col) { **if** (mask.at<uchar>(row, col) > 0) { *//so this pixel needs to be filled* colorImage.at<cv::Vec3b>(row,col)= cv::Vec3b(colorImage.at<cv::Vec3b>(label_to_index[labels.at<**int**>(row, col)])[0], colorImage.at<cv::Vec3b>(label_to_index[labels.at<**int**>(row, col)])[1], colorImage.at<cv::Vec3b>(label_to_index[labels.at<**int**>(row, col)])[2]); } } }

**Figure 11:**
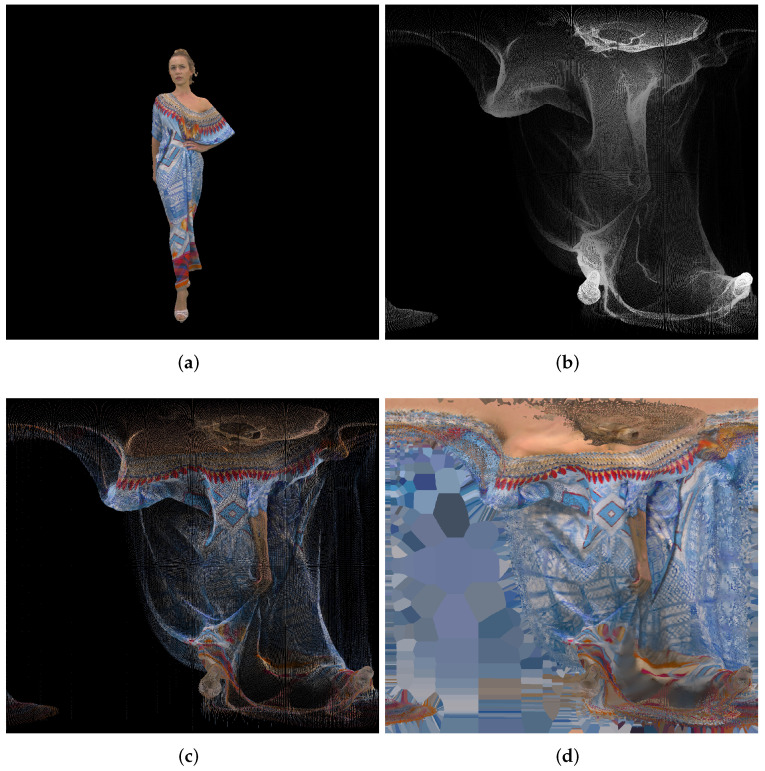
Point clouds to panorama, using Miller projection: (**a**) original point cloud “longdress_vox10_1060.ply” (799,765 points); (**b**) geometry (1496 × 1344 pixels, 16-bit grayscale); (**c**) texture (1496 × 1344 pixels, 24-bit RGB); (**d**) texture with Voronoi (1496 × 1344 pixels, 24-bit RGB).

The previously described algorithm is presented in Figure 11, for the tenth point cloud “longdress_vox10_1060.ply” and the Miller projection type. The projection area is set to be about 2,000,000 pixels. We define the ratio for the image size as the ratio between the first point cloud height and width, and again divided by π. In this example case, this ratio is 0.8968. Because later video compression expects frame width and height divisible by 8, we also round the final frame width and height to the nearest integer divisible by 8, Equation (23). Again, for the later video compression, all subsequent point clouds need to have the same frame size.

(23) $$\begin{array}{cc} \; & \mathrm{ratio}=\frac{\mathrm{pc}\_\mathrm{height}}{\pi \cdot \mathrm{pc}\_\mathrm{width}}\\ \; & \mathrm{frame}\_\mathrm{width}=8\cdot \left\lfloor \left(\frac{1}{8}\cdot \sqrt{\frac{\mathrm{panorama}\_\mathrm{area}}{\mathrm{ratio}}}\right)\right\rceil \\ \; & \mathrm{frame}\_\mathrm{height}=8\cdot \left\lfloor \left(\frac{1}{8}\cdot \mathrm{frame}\_\mathrm{width}\cdot \mathrm{ratio}\right)\right\rceil \end{array}$$

After the first point cloud has been projected to 2D panorama, all the other subsequent point clouds are also projected to the panorama for geometry and texture with the same frame size as the first one. Also, geometry and texture are calculated in the same way as earlier described. Afterwards, we use FFmpeg and different video compressions for geometry and texture images. For texture, we use the x265 coder (H.265/HEVC) with lossy compression, while for geometry we use the FFV1 coder with lossless compression. For x265 (for texture) we use crf 17 (constant rate factor), pixel format rgb24, preset veryslow, while for FFV1 (for geometry) we use pixel format gray9le (depth of 9 bits per pixel), as well as gray10le (only in the case of Miller projection). For this case, the texture file size is $11.3\times 10^{6}$ bytes and the geometry file size is $14\times 10^{6}$ bytes, so overall size is $25.3\times 10^{6}$ bytes.

Point cloud recreation is done by decompressing previously compressed video files, and afterwards using inverse formulas for used projection type, in this example Miller. For the color information, we use pixels from the panorama image for color (Voronoi diagram) that exist in panorama image for geometry. In the final step, we use several algorithms to oversample the original point cloud and fill holes that may exist in some parts of it:

- normal estimation using CloudCompare version 2.11.1 x64 [46], for the later screened Poisson reconstruction, in “command line” mode. Specific parameters used were: -OCTREE_NORMALS auto -ORIENT PLUS_ZERO -MODEL TRI -ORIENT_NORMS_MST 8 -ORIENT_NORMS_MST 4.
- Surface Reconstruction: Screened Poisson filter using MeshLab 2020.09 [45]: reconstruction depth 11 and other default values.
- Poisson-disk Sampling filter using MeshLab: using the same number of samples as the original point cloud, Monte Carlo OverSampling of 20 and other default values. However, usually somewhat higher number of output points were created.
- Vertex Attribute Transfer filter using MeshLab: using default values.

**Figure 12:**
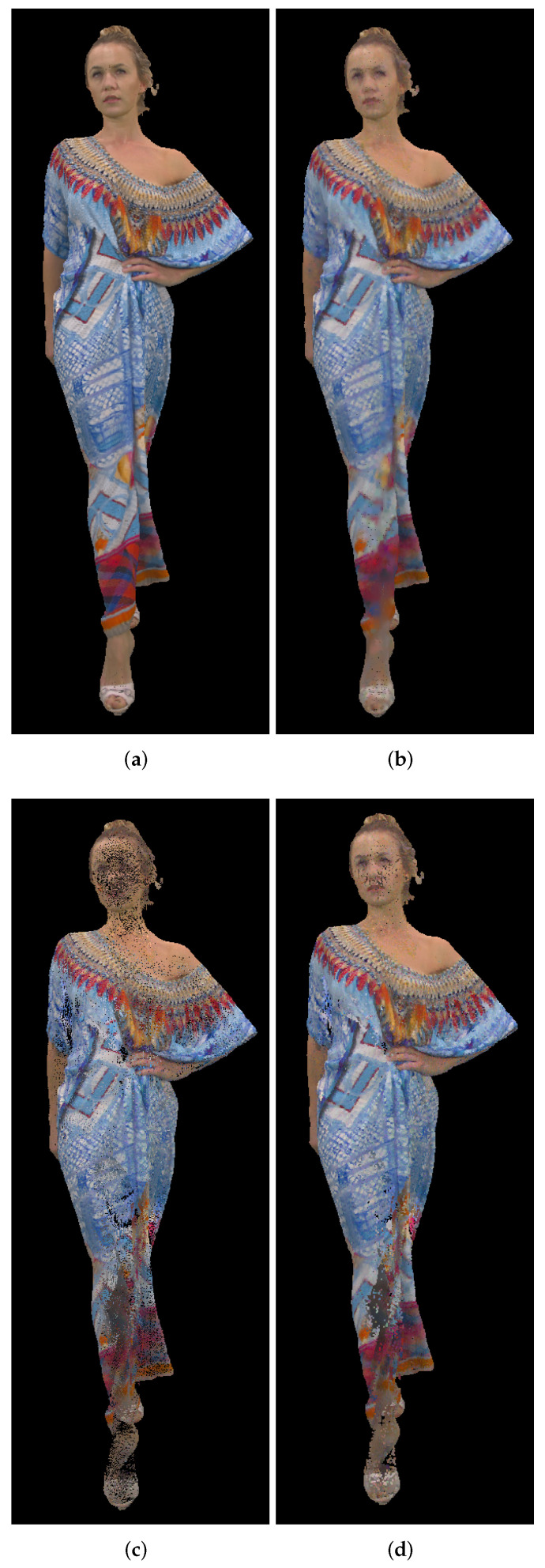
Point clouds to panorama, using Miller projection and 9-bit range depth: (**a**) Original point cloud “longdress_vox10_1060.ply” (799,765 points; point size 1 in MeshLab); (**b**) Decompressed point cloud after Poisson reconstruction (1,161,124 points; point size 1 in MeshLab); (**c**) Decompressed point cloud before Poisson reconstruction (430,793 points; point size 1 in MeshLab); (**d**) Decompressed point cloud before Poisson reconstruction (430,793 points; point size 2 in MeshLab).

After the point cloud has been recreated, we need to scale it to its initial size and also translate it to its original position. Those 4 numbers (1 float for scaling and 3 floats for translation in each direction) have to be transmitted as well. Original point cloud snapshot and point cloud snapshots before and after the Poisson reconstruction algorithm are presented in Figure 12.

Tested 20 input point clouds have overall 15,900,190 points, on average 795,010 points per point cloud, while the overall compressed file size is $25.3\times 10^{6}$ B. In this case we use approximately $25.3\times 10^{6}\times 8/\left(20\times 795,010\right)=12.7$ bits per (input) point, or, if compared with the output number of points, we use approximately $25.3\times 10^{6}\times 8/\left(20\times 1,154,324\right)=8.8$ bits per output point. However, the Poisson reconstruction algorithm at the end of the decompression may produce even higher number of output points (in this case average number of points is 1,154,324), than the input number of points, so in this case the number of bits per output point may be misleading. Because of that, later we report only bits per input point. Poisson reconstruction step is an important part of the proposed algorithm, cf. Figure 12. Used scripts can be found on the web page [49].

## 5. Results

### 5.1. Objective Measures Used for Point Cloud Performance Comparison

Recently, several objective measures have been proposed, based on geometry or/and attribute information of the tested point clouds [50]. For geometry, two different groups of measures have been proposed: point-to-point (*p2p*) and point-to-plane distances (*p2pl*) [51]. Later in the paper, as point-to-point measures we will use rmsFp2p, ${\mathrm{rmsFPSNR}}_{1}\;p2p$ and ${\mathrm{rmsFPSNR}}_{2}\;p2p$ measures defined as:

(24) $$\mathrm{rmsF}\;p2p=\sqrt{\frac{1}{n}\sum_{i=1}^{n}\left|\right|E_{i,j}{\left|\right|}_{2}^{2}}$$

(25) $${\mathrm{rmsFPSNR}}_{1}\;p2p=10\log_{10}\left(\frac{\mathrm{MAX}\_\mathrm{DIST}}{{\left(\mathrm{rmsF}\;p2p\right)}^{2}}\right)$$

(26) $${\mathrm{rmsFPSNR}}_{2}\;p2p=10\log_{10}\left(\frac{3c^{2}}{{\left(\mathrm{rmsF}\;p2p\right)}^{2}}\right),c=2^{d}-1$$

In Equation (24), $E_{i,j}$ is defined as the difference vector (or point to nearest point vector) between an arbitrary point from the first point cloud and the corresponding nearest point from the second point cloud. The first and second point clouds are firstly original and degraded and then vice versa. Final (symmetric) measure is calculated as the measure with worse (higher rms) score.

In Equation (25) MAX_DIST is maximal distance between all pairs of closest points in the first and second point cloud, as defined in [51]. In Equation (26) *c* is the peak constant value, depending on the point cloud coordinates precision *d* (e.g., d=10 for 10-bit depth precision), as used in [19] during the development of the MPEG standard.

Similar to the point-to-point measures, point-to-plane measures are defined as:

(27) $$\mathrm{rmsF}\;p2\mathrm{pl}=\sqrt{\frac{1}{n}\sum_{i=1}^{n}{\left(<E_{i,j},N_{j}>\right)}^{2}}$$

(28) $${\mathrm{rmsFPSNR}}_{1}\;p2p=10\log_{10}\left(\frac{\mathrm{MAX}\_\mathrm{DIST}}{{\left(\mathrm{rmsF}\;p2\mathrm{pl}\right)}^{2}}\right)$$

(29) $${\mathrm{rmsFPSNR}}_{2}\;p2p=10\log_{10}\left(\frac{3c^{2}}{{\left(\mathrm{rmsF}\;p2\mathrm{pl}\right)}^{2}}\right),c=2^{d}-1$$

In Equation (27) $E_{i,j}$ is defined similarly as for the *p2p* measures. $N_{j}$ is the unit normal vector, calculated for each point in the first point cloud. $<E_{i,j},N_{j}>$ is the dot product between error vector $E_{i,j}$ and normal vector $N_{j}$, obtaining projected error vector. In Equation (28) parameter MAX_DIST is the same as in Equation (25) and in Equation (29) parameters *c* and *d* are the same as in the Equation (26).

In the next subsection, point-to-point and point-to-plane measures will be calculated using the software presented in [51].

### 5.2. Point Cloud Compression Using Different Projections—Compression Efficiency

**Figure 13:**
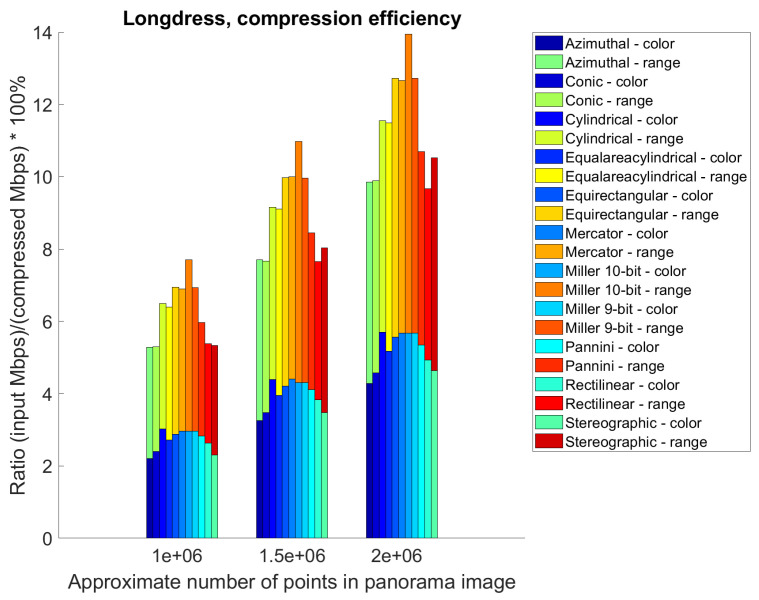
Average ratio of the input file sizes and the color and range compressed video file sizes, created from the panorama images, using point cloud *Longdress*. Ratio is presented for all cases, e.g., for 1,000,000, 1,500,000 and 2,000,000 points.

Table 1, Table 2 and Table 3 present a basic overview of the compression efficiency of point clouds with different projection area: 1056 × 944 pixels (approximately 1,000,000), 1296 × 1160 pixels (approximately 1,500,000) and 1496 × 1344 pixels (approximately 2,000,000) respectively. Separately, we calculated bpp (bits per input point) and Mbps (bpp × average number of input points per point cloud × 30 point clouds per second ×$10^{-6}$) for color, range and color+range compressed video files. Results from all three tables are also summarized in the Figure 13, which represents average ratio of the input file sizes and the color and range compressed video file sizes, created from the panorama images, using point cloud *Longdress*. Higher ratio does not represent better case (i.e., better objective scores), but only higher number of used bits per input point, for the same panorama size. It can be concluded that equirectangular, Miller and Mercator projections have the highest bpp for the tested point cloud *Longdress*.

### 5.3. Point Cloud Compression Using Different Projections—Objective Measures

**Figure 14:**
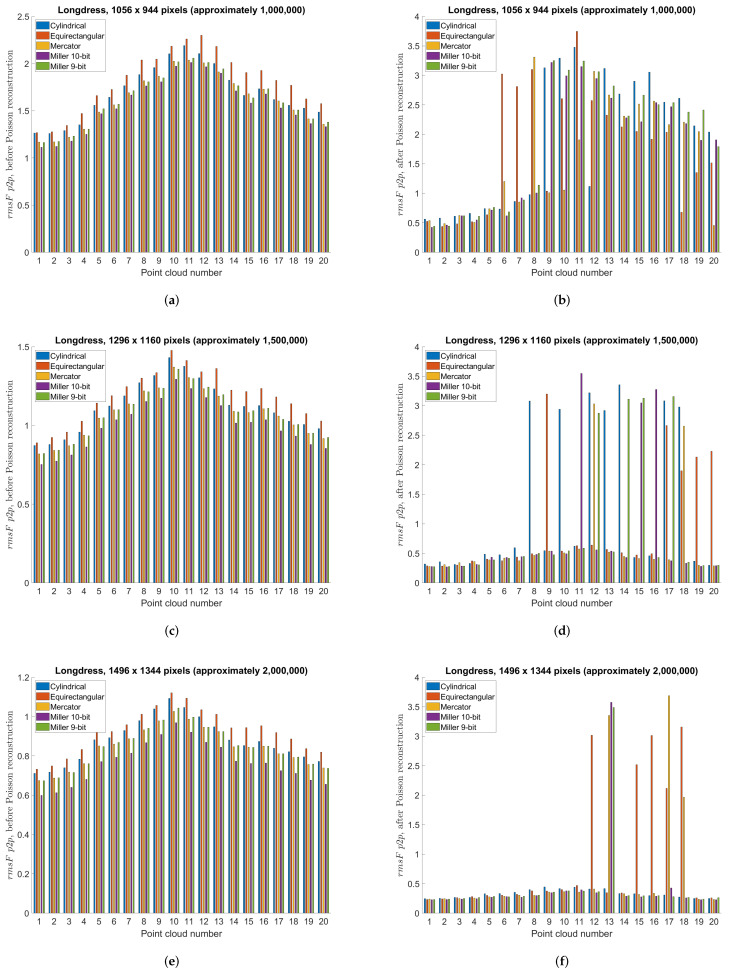
Point-to-point objective measure per point cloud, using cylindrical, equirectangular, Mercator, Miller 10-bit and Miller 9-bit projections: (**a**) before Poisson reconstruction, projection area 1056 × 944 pixels (approximately 1,000,000); (**b**) after Poisson reconstruction, projection area 1056 × 944 pixels (approximately 1,000,000); (**c**) before Poisson reconstruction, projection area 1296 × 1160 pixels (approximately 1,500,000); (**d**) after Poisson reconstruction, projection area 1296 × 1160 pixels (approximately 1,500,000); (**e**) before Poisson reconstruction, projection area 1496 × 1344 pixels (approximately 2,000,000); (**f**) after Poisson reconstruction, projection area 1496 × 1344 pixels (approximately 2,000,000).

**Figure 15:**
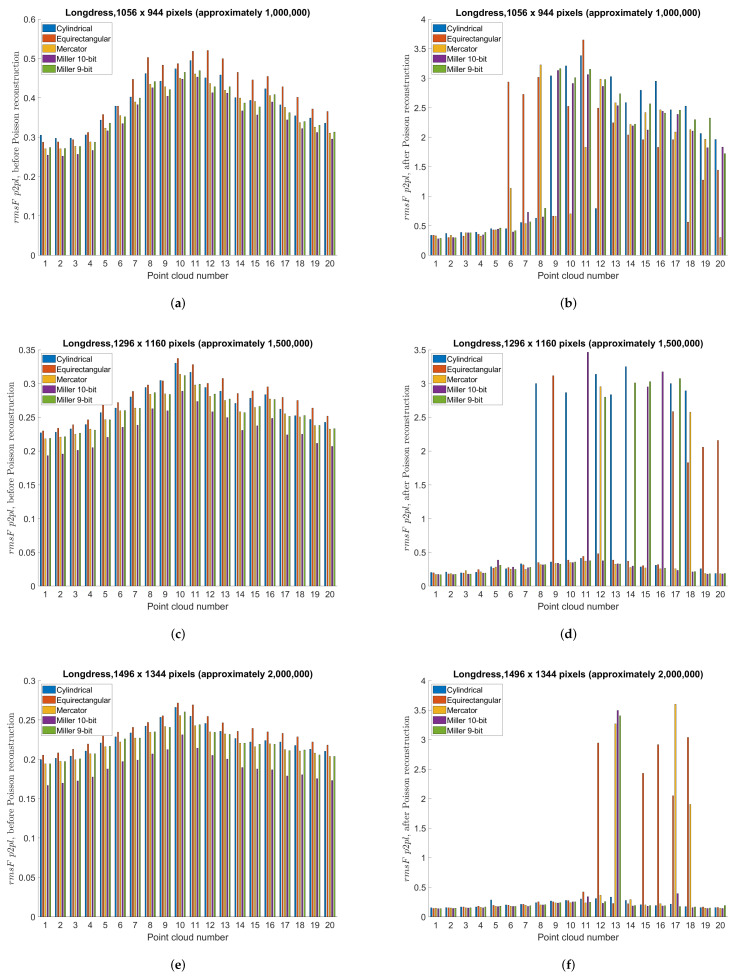
Point-to-plane objective measure per point cloud, using cylindrical, equirectangular, Mercator, Miller 10-bit and Miller 9-bit projections: (**a**) before Poisson reconstruction, projection area 1056 × 944 pixels (approximately 1,000,000); (**b**) after Poisson reconstruction, projection area 1056 × 944 pixels (approximately 1,000,000); (**c**) before Poisson reconstruction, projection area 1296 × 1160 pixels (approximately 1,500,000); (**d**) after Poisson reconstruction, projection area 1296 × 1160 pixels (approximately 1,500,000); (**e**) before Poisson reconstruction, projection area 1496 × 1344 pixels (approximately 2,000,000); (**f**) after Poisson reconstruction, projection area 1496 × 1344 pixels (approximately 2,000,000).

**Figure 16:**
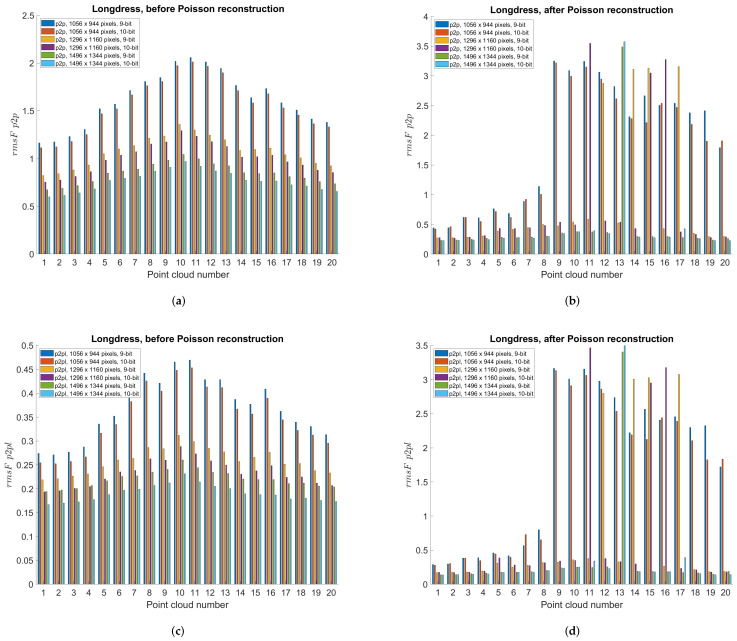
Objective measure per point cloud, using Miller projection with 9-bit depth and 10-bit depth: (**a**) before Poisson reconstruction, point-to-point measure; (**b**) after Poisson reconstruction, point-to-point measure; (**c**) before Poisson reconstruction, point-to-plane measure; (**d**) after Poisson reconstruction, point-to-plane measure.

This section compares different projection methods with three different panorama sizes using previously described point-to-point and point-to-plane objective measures. Specifically, Table 4, Table 5 and Table 6 present objective measures before Poisson reconstruction, while Table 7, Table 8 and Table 9 present objective measures after Poisson reconstruction. Best values are bolded in all tables. Figure 14 presents point-to-point measure separately per each point cloud, for cylindrical, equirectangular, Mercator, Miller 10-bit and Miller 9-bit projections, before and after Poisson reconstruction, for all three panorama sizes, while Figure 15 similarly presents point-to-plane measure. Finally, Figure 16 compares 9-bit and 10-bit Miller projections using point-to-point and point-to-plane measures, before and after Poisson reconstruction.

### 5.4. Point Cloud Compression—Timing Performance

In this section we will present timing performance for the case of Miller 9-bit projection with panorama size of 2,000,000 points, Table 10. Point cloud to panorama and vice versa timing is averaged across 20 point clouds, while for video compression and decompression we use FFmpeg and different video compressions for geometry and texture images described earlier. For texture, we use the x265 coder (H.265/HEVC) with lossy compression, while for geometry we use the FFV1 coder with lossless compression. For x265 (for texture) we use crf 17 (constant rate factor), pixel format rgb24, preset veryslow, while for FFV1 (for geometry) we use pixel format gray9le (depth of 9 bits per pixel). Computer performance is: Intel i7-4770 @ 3.40 GHz, 16 GB RAM, using virtual machine on Windows 7 x64, running Ubuntu x64 18.04 LTS. It can be seen that Poisson reconstruction and upsampling process takes most of the overall time, compared with other steps. Minor steps in between presented were not taken into account (creating readable point clouds for CloudCompare and Meshlab by adding header in created point clouds from 3DTK and copying files). Due to the overall timing, we did not test all 300 point clouds in a *Longdress* sequence, but we used first 20 point clouds. However, we expect similar performance for the other point clouds as well.

### 5.5. Point Cloud Compression Using Different Point Clouds

**Figure 17:**
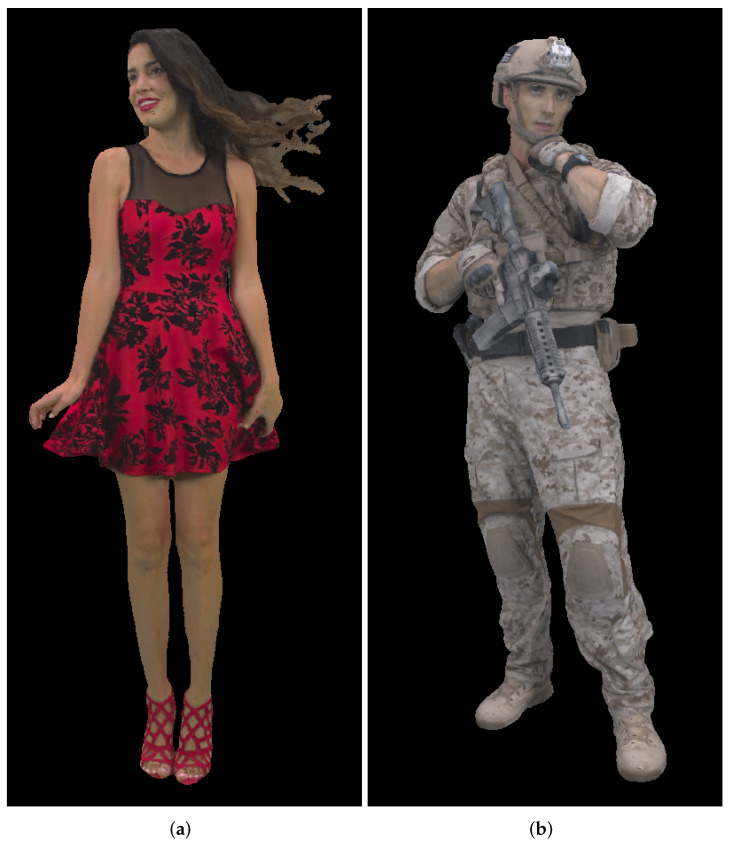
Tested point clouds: (**a**) *Redandblack*, “redandblack_vox10_1450.ply”; (**b**) *Soldier*, “soldier_vox10_0536.ply”.

**Figure 18:**
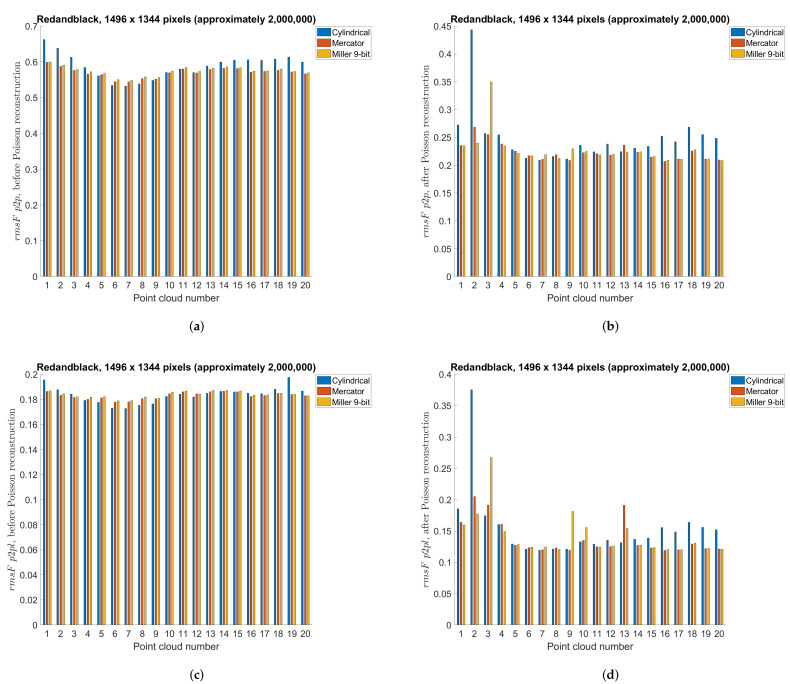
Point-to-point and point-to-plane objective measure (rmsFp2p) per *Redandblack* point cloud, using cylindrical, Mercator and Miller 9-bit depth projections with approximately 2,000,000 points: (**a**) point-to-point, before Poisson reconstruction; (**b**) point-to-point, after Poisson reconstruction; (**c**) point-to-plane, before Poisson reconstruction; (**d**) point-to-plane, after Poisson reconstruction.

**Figure 19:**
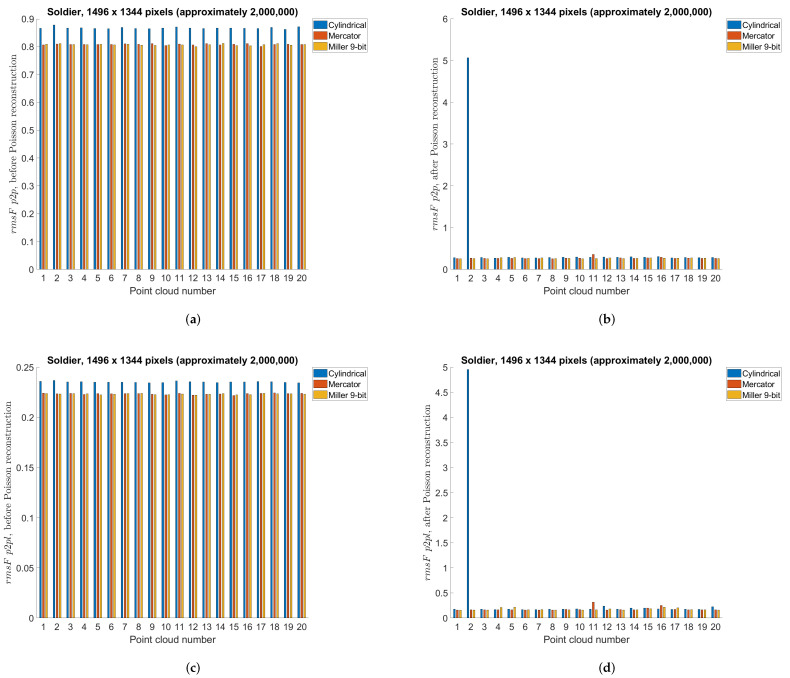
Point-to-point objective measure (rmsFp2p) per *Soldier* point cloud, using cylindrical, Mercator and Miller 9-bit depth projections with approximately 2,000,000 points: (**a**) point-to-point, before Poisson reconstruction; (**b**) point-to-point, after Poisson reconstruction; (**c**) point-to-plane, before Poisson reconstruction; (**d**) point-to-plane, after Poisson reconstruction.

In this section we will present results for two different dynamic point clouds, *Redandblack* and *Soldier* (Figure 17) from the dynamic point cloud dataset [44]. Similar to the previous test cases, we used 20 point clouds from the beginning (“redandblack_vox10_1450.ply”–“redandblack_vox10_1469.ply” and “soldier_vox10_0536.ply” – “soldier_vox10_0555.ply”). Panorama image size of approximately 2,000,000 points and cylindrical, Mercator and Miller 9-bit projection types have been used. Results for the point-to-point and point-to-plane measures are shown in Figure 18 for *Redandblack* and Figure 19 for *Soldier* point clouds.

**Figure 20:**
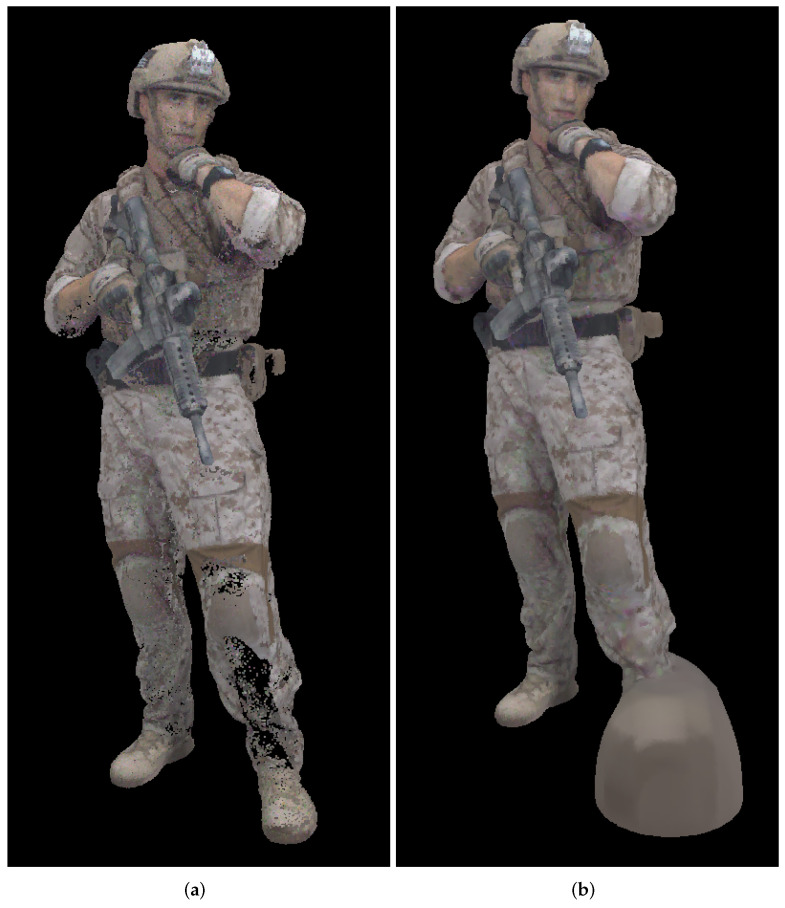
Decompressed point cloud *Soldier*, “soldier_vox10_0537.ply”-second point cloud used in compression/decompression process, using cylindrical projection with panorama size of 2,000,000 points: (**a**) before Poisson reconstruction (point size 2 in MeshLab); (**b**) after Poisson reconstruction (point size 1 in MeshLab).

Figure 20 shows second decompressed point cloud *Soldier*, using cylindrical projection with panorama size of 2,000,000 points, “soldier_vox10_0537.ply”. In Figure 20a we don’t use Poisson reconstruction, while in Figure 20b we use Poisson reconstruction algorithm described earlier. It can be seen that in Figure 20a there are missing points on the left leg, which creates wrongly oriented normals and in Figure 20b there exist an artifact, not present in the original point cloud. Due to this error, there is a noticeable increase (lower quality) of point-to-point and point-to-plane measures, as it can be seen in Figure 19b,d, for the point cloud number 2 and cylindrical projection (blue color).

### 5.6. Comparison with Octree Reduction from 3DTK Toolkit

In this section we will present results using 3DTK toolkit and octree reduction method, for the previously tested point clouds: *Longdress* (Table 11 and Table 12), *Redandblack* (Table 13 and Table 14) and *Soldier* (Table 15 and Table 16). Generally, parameter “R” turns on octree based point reduction with voxels of size $R^{3}$, while parameter “O” enables randomized octree based point reduction with O points per voxel. We used “scan_red” and “show” programs from 3DTK toolkit to create reduced point clouds: “scan_red” to create decompressed octree-based reduced point clouds and “show” to create compressed .oct files. With increasing “R” we create lower number of points, while with increasing “O” we create higher number of output points. Decompressed point clouds have been compared with the original point clouds using previously described point-to-point and point-to-plane metrics. Average size of output .oct file is also reported, as well as their bits per input point (bpp).

### 5.7. Discussion

From Table 4, Table 5 and Table 6 e.g., before the Poisson surface reconstruction algorithm, the best projection is Miller 10-bit, however Miller 9-bit and Mercator (also 9-bit) are similarly performed. Probably because of the higher bit depth, Miller 10-bit is here the best projection, however 1 extra bit may not be justified by only a little better objective measures, Figure 16. From Table 7, Table 8 and Table 9 e.g., after the Poisson reconstruction algorithm, Mercator projection is the best in the cases with projection areas of 1056 × 944 pixels and 1296 × 1160 pixels, while cylindrical projection is the best for the projection area of 1496 × 1344 pixels. In the case of the bigger projection area, there are more points, so surface reconstruction algorithm gives better results. Actually, the main problem is the automatic calculation of normal vectors: in some cases, inverted normal vectors are calculated so Poisson reconstruction algorithm afterwards creates unwanted artifacts, lowering the overall objective score rmsF. If the point clouds originate from sensor measurements, then the sensor poses enable a consistent orientation of the normal vectors. Artifacts from inconsistent normals are noticable in Figure 14 and Figure 15 as higher scores in those point clouds. It can be seen that in the best case, e.g., cylindrical projection with a projection area of 1496 × 1344 pixels, Figure 15f, there are no unexpected errors, so the average score is the best for this case. The Miller projection also gives very good results, with only 1 larger error for the largest projection area. However, with smaller projection areas, some of the points in the original point cloud become occluded by other points, so that they are not visible in the used projection. Because of that, larger holes appear in the decompressed point cloud, which makes it difficult to calculate normal vectors and finally surface reconstruction. This can be seen especially in the smallest projection area that was tested, with a size 1056 × 944 pixels. In this case, average rmsF objective score measures are the same before and after surface reconstruction, meaning that the results were not better with the reconstruction algorithm. From Figure 18, *Redandblack* point cloud, all proposed projections generally give good results with Poisson reconstruction. From Figure 19, *Soldier* point cloud, Mercator and Miller projections give good results with Poisson reconstruction, while cylindrical projection creates one error for the second point cloud. Possibly, newer projection maps might be used, for example as in the irregularly shaped objects in astronomy [52].

In Equation (22) we are using 16-bit precision, newly added in 3DTK toolkit, so that in this paper we have the best representation for 16-bit precision, which can be also saved in png file and stored using 3DTK toolkit (which uses OpenCV to store images). However, later we are compressing geometry using 9-bit (or 10-bit for Miller compression) and FFV1 video compression. FFV1 decoder also creates 16-bit png images, but with maximally $2^{9}$ (or $2^{10}$) different intensities. This is different, compared to the reference [9], because in the reference [9] we used only 24-bit geometry precision, before video compression. Compared with the reference [9], similar parameters were for 1920x1080 resolution and equirectangular projection, where 64,160,627 bytes (for the geometry and color) were used, while in this paper for panorama size of 2,000,000 and equirectangular projection we use 12.7261 bits per point or (multiplied by 795,010 average number of input points and 20 point clouds) 25,293,426 bytes, which is 39.4% from [9]. Separately, in [9] we use 21,378,775 bytes for the geometry, while in this paper we use 14,225,893 bytes or 66.5% from [9]. For the color, in [9] we use 43,208,605 bytes, while in this paper we use 11,067,533 bytes or 25.6% from [9]. Also, somewhat better pixel occupancy is achieved in this paper, compared to the reference [9]: for the same case (and equirectangular projection), average decompressed point cloud Longdress has 428,221 points (before Poisson reconstruction) in this paper and 398,905 in [9]. This might be also compared with equirectangular projection and panorama size of 1,500,000 points, where average decompressed point cloud has 390,058 points (before Poisson reconstruction). For this case, we use 9.9778 bits per point or 19,831,040 bytes. which is 30.9% from [9]. Better projection methods, like Miller, gives for the same panorama size (1,500,000 points) on average higher number of output point cloud points—406,868 and also needs 19,789,828 bytes for the geometry and color, or 30.8% from [9]. In the reference [10], larger static point clouds were also used with bigger range dynamic, which might need 24-bit representation. However, in this paper we used only voxelized dynamic point clouds with the size 1024×1024×1024, so 9-bit representation might be enough.

In comparison with the results from octree reduction, the proposed solution gives better results for a similar size. However, the octree reduction algorithm has not been designed for dynamic point clouds, and it was designed for other types of point clouds, such as point clouds created by LIDAR, with non-uniform density and sampling, with much higher bit-depth, in which case results might be different. Also, in octree-based reduction, points do not occlude each other, so it creates a uniformly (sub)sampled point cloud, independent of the final number of points, compared to the proposed solution.

## 6. Conclusions

In this paper, we have proposed a new projection-based point cloud compression using different projection types, video compression algorithms, and surface reconstruction algorithm. Ten different projection types and three different projection area sizes have been considered, and objective point-to-point and point-to-plane measures were calculated. The results showed that, overall, the Miller projection can be considered as the best among the tested projections. The Mercator projection needs to be modified to address the problem of representing latitudes φ near $\pm 90^{\circ }$. Cylindrical projection has worse objective scores for smaller panorama size (among tested sizes). Also, although the Poisson surface reconstruction can imply some artifacts due to the missing points in the raw decompressed point cloud, it is an important step of the proposed point cloud compression which fills the missing points and usually generates better visual quality of reconstructed point clouds, at least for larger panorama sizes and tested point clouds.

Future research will consider different projection types which may keep points without creating larger holes and better algorithms for normal vector calculation which may provide higher compression ratios. Different empty-pixel color filling methods might also be considered in future research, such as horizontal filling used by MPEG’s V-PCC compression.

## Figures and Tables

**Table 1:** Information about the compressed file sizes for different projection types, with projection area of 1056 × 944 pixels (approximately 1,000,000).

1056 × 944 Pixels (Approximately 1,000,000)	Azimuthal	Conic	Cylindrical	Equalareacylindrical	Equirectangular	Mercator	Miller 10-Bit	Miller 9-Bit	Pannini	Rectilinear	Stereographic
Compressed color average bits per input point (bpp):	2.2018	2.4025	3.0207	2.7203	2.8726	2.9612	2.9618	2.9618	2.826	2.63	2.3026
Compressed range average bits per input point (bpp):	3.0784	2.8992	3.4701	3.6785	4.074	3.9306	4.7362	3.9652	3.1382	2.7561	3.0313
Compressed range+color average bits per input point (bpp):	5.2803	5.3016	6.4908	6.3987	6.9467	6.8918	7.698	6.927	5.9642	5.386	5.3339
Input average bits per input point (binary format) (bpp):	120.0017	120.0017	120.0017	120.0017	120.0017	120.0017	120.0017	120.0017	120.0017	120.0017	120.0017
Ratio (input bpp)/(compressed bpp) * 100%	4.4001	4.418	5.409	5.3322	5.7888	5.7431	6.4149	5.7724	4.9701	4.4883	4.4448
Compressed video color (Mbps):	52.514	57.2993	72.0449	64.8792	68.5135	70.626	70.6387	70.6387	67.3999	62.7257	54.9167
Compressed video range (Mbps):	73.4217	69.146	82.7638	87.7325	97.1667	93.7467	112.9608	94.5713	74.8475	65.7331	72.2982
Compressed video range+color (Mbps):	125.9357	126.4452	154.8087	152.6117	165.6802	164.3727	183.5995	165.2101	142.2474	128.4588	127.2149
Input point cloud (binary format) (Mbps):	2862.0774	2862.0774	2862.0774	2862.0774	2862.0774	2862.0774	2862.0774	2862.0774	2862.0774	2862.0774	2862.0774
Ratio (input Mbps)/(compressed Mbps) * 100%	4.4001	4.4180	5.4090	5.3322	5.7888	5.7431	6.4149	5.7724	4.9701	4.4883	4.4448

**Table 2:** Information about the compressed file sizes for different projection types, with projection area of 1296 × 1160 pixels (approximately 1,500,000).

1296 × 1160 Pixels (Approximately 1,500,000)	Azimuthal	Conic	Cylindrical	Equalareacylindrical	Equirectangular	Mercator	Miller 10-Bit	Miller 9-Bit	Pannini	Rectilinear	Stereographic
Compressed color average bits per input point (bpp):	3.2534	3.4712	4.3975	3.9488	4.2032	4.4046	4.3085	4.3085	4.1135	3.8306	3.4746
Compressed range average bits per input point (bpp):	4.4431	4.1957	4.7533	5.1534	5.7746	5.5972	6.6708	5.6485	4.3288	3.8173	4.5586
Compressed range+color average bits per input point (bpp):	7.6965	7.6669	9.1508	9.1022	9.9778	10.0018	10.9793	9.957	8.4423	7.6479	8.0332
Input average bits per input point (binary format) (bpp):	120.0017	120.0017	120.0017	120.0017	120.0017	120.0017	120.0017	120.0017	120.0017	120.0017	120.0017
Ratio (input bpp)/(compressed bpp) * 100%	6.4136	6.389	7.6255	7.585	8.3147	8.3347	9.1493	8.2974	7.0352	6.3732	6.6942
Compressed video color (Mbps):	77.5949	82.7901	104.881	94.18	100.2465	105.0509	102.7599	102.7599	98.109	91.36	82.8696
Compressed video range (Mbps):	105.9685	100.0686	113.3672	122.9091	137.726	133.4953	159.1008	134.7181	103.2435	91.0447	108.7247
Compressed video range+color (Mbps):	183.5634	182.8587	218.2482	217.0891	237.9725	238.5462	261.8607	237.4779	201.3525	182.4047	191.5943
Input point cloud (binary format) (Mbps):	2862.0774	2862.0774	2862.0774	2862.0774	2862.0774	2862.0774	2862.0774	2862.0774	2862.0774	2862.0774	2862.0774
Ratio (input Mbps)/(compressed Mbps) * 100%	6.4136	6.3890	7.6255	7.5850	8.3147	8.3347	9.1493	8.2974	7.0352	6.3732	6.6942

**Table 3:** Information about the compressed file sizes for different projection types, with projection area of 1496 × 1344 pixels (approximately 2,000,000).

1496 ×1344 Pixels (Approximately 2,000,000)	Azimuthal	Conic	Cylindrical	Equalareacylindrical	Equirectangular	Mercator	Miller 10-Bit	Miller 9-Bit	Pannini	Rectilinear	Stereographic
Compressed color average bits per input point (bpp):	4.2758	4.5804	5.6975	5.1732	5.5685	5.675	5.669	5.669	5.3434	4.9298	4.6411
Compressed range average bits per input point (bpp):	5.5811	5.3118	5.8544	6.3141	7.1576	6.9908	8.2718	7.0543	5.348	4.7428	5.8841
Compressed range+color average bits per input point (bpp):	9.8569	9.8922	11.5519	11.4873	12.7261	12.6657	13.9409	12.7233	10.6913	9.6726	10.5252
Input average bits per input point (binary format) (bpp):	120.0017	120.0017	120.0017	120.0017	120.0017	120.0017	120.0017	120.0017	120.0017	120.0017	120.0017
Ratio (input bpp)/(compressed bpp) * 100%	8.2139	8.2434	9.6265	9.5726	10.6049	10.5546	11.6172	10.6026	8.9093	8.0604	8.7709
Compressed video color (Mbps):	101.9793	109.2427	135.8868	123.3824	132.8104	135.3498	135.2079	135.2079	127.4406	117.5775	110.6906
Compressed video range (Mbps):	133.1103	126.6887	139.6304	150.594	170.7107	166.7318	197.286	168.2461	127.5504	113.1165	140.3382
Compressed video range+color (Mbps):	235.0896	235.9314	275.5172	273.9764	303.5211	302.0816	332.4939	303.454	254.991	230.6941	251.0288
Input point cloud (binary format) (Mbps):	2862.0774	2862.0774	2862.0774	2862.0774	2862.0774	2862.0774	2862.0774	2862.0774	2862.0774	2862.0774	2862.0774
Ratio (input Mbps)/(compressed Mbps) * 100%	8.2139	8.2434	9.6265	9.5726	10.6049	10.5546	11.6172	10.6026	8.9093	8.0604	8.7709

**Table 4:** Point-to-point (p2p) and point-to-plane (p2pl) objective measures rmsF, ${\mathrm{rmsFPSNR}}_{1}$ and ${\mathrm{rmsFPSNR}}_{2}$, before Poisson reconstruction, using different projections with projection area of 1056 × 944 pixels (approximately 1,000,000), best values are bold; average input and output number of points and their ratio.

	rmsFp2p	${\mathrm{rmsFPSNR}}_{1}\;p2p$	${\mathrm{rmsFPSNR}}_{2}\;p2p$	rmsFp2pl	${\mathrm{rmsFPSNR}}_{1}\;p2\mathrm{pl}$	${\mathrm{rmsFPSNR}}_{2}\;p2\mathrm{pl}$	Average Input Points	Average Output Points	Output/Input·100%
Azimuthal	4.3569	58.5848	−5.7881	1.1374	64.4248	5.8920	795,010	260,522	32.7697
Conic	3.8489	59.1248	−4.7080	1.0048	64.9778	6.9979	795,010	250,454	31.5033
Cylindrical	1.6901	62.7506	2.5435	0.3878	69.1378	15.3180	795,010	318,703	40.0879
Equalareacylindrical	3.2290	59.8909	−3.1758	0.7819	66.0874	9.2171	795,010	283,737	35.6897
Equirectangular	1.8139	62.4501	1.9426	0.4156	68.8628	14.7679	795,010	333,865	41.9951
Mercator	1.6177	62.9459	2.9342	0.3677	69.3777	15.7978	795,010	344,682	43.3557
Miller 10-bit	**1.5704**	**63.0796**	**3.2017**	**0.3505**	**69.5962**	**16.2347**	795,010	345,223	43.4237
Miller 9-bit	1.6200	62.9388	2.9200	0.3686	69.3682	15.7787	795,010	345,223	43.4237
Pannini	1.7741	62.5377	2.1177	0.4092	68.9031	14.8485	795,010	291,890	36.7153
Rectilinear	1.9423	62.1427	1.3278	0.4655	68.3559	13.7542	795,010	260,368	32.7503
Stereographic	3.6261	59.3823	−4.1930	0.8135	65.9116	8.8655	795,010	283,551	35.6663

**Table 5:** Point-to-point (p2p) and point-to-plane (p2pl) objective measures rmsF, ${\mathrm{rmsFPSNR}}_{1}$ and ${\mathrm{rmsFPSNR}}_{2}$, before Poisson reconstruction, using different projections with projection area of 1296 × 1160 pixels (approximately 1,500,000), best values are bold; average input and output number of points and their ratio.

	rmsFp2p	${\mathrm{rmsFPSNR}}_{1}\;p2p$	${\mathrm{rmsFPSNR}}_{2}\;p2p$	rmsFp2pl	${\mathrm{rmsFPSNR}}_{1}\;p2\mathrm{pl}$	${\mathrm{rmsFPSNR}}_{2}\;p2\mathrm{pl}$	Average Input Points	Average Output Points	Output/Input·100%
Azimuthal	3.4281	59.6300	−3.6976	0.8209	65.8651	8.7725	795,010	313,372	39.4174
Conic	3.0146	60.1911	−2.5754	0.7793	66.0917	9.2258	795,010	300,309	37.7742
Cylindrical	1.1212	64.5158	6.0740	0.2697	70.6845	18.4113	795,010	389,681	49.0159
Equalareacylindrical	2.4257	61.1371	−0.6835	0.5808	67.3735	11.7893	795,010	329,721	41.4738
Equirectangular	1.1851	64.2722	5.5868	0.2797	70.5251	18.0925	795,010	390,058	49.0633
Mercator	1.0756	64.6961	6.4347	0.2590	70.8577	18.7578	795,010	406,907	51.1826
Miller 10-bit	**1.0077**	**64.9852**	**7.0127**	**0.2333**	**71.3168**	**19.6760**	795,010	406,868	51.1777
Miller 9-bit	1.0758	64.6942	6.4308	0.2593	70.8521	18.7466	795,010	406,868	51.1777
Pannini	1.1853	64.2675	5.5774	0.2849	70.4427	17.9277	795,010	359,861	45.2650
Rectilinear	1.3303	63.7622	4.5667	0.3185	69.9618	16.9659	795,010	324,548	40.8231
Stereographic	2.5708	60.8974	−1.1628	0.5539	67.6207	12.2838	795,010	343,921	43.2600

**Table 6:** Point-to-point (p2p) and point-to-plane (p2pl) objective measures rmsF, ${\mathrm{rmsFPSNR}}_{1}$ and ${\mathrm{rmsFPSNR}}_{2}$, before Poisson reconstruction, using different projections with projection area of 1496 × 1344 pixels (approximately 2,000,000), best values are bold; average input and output number of points and their ratio.

	rmsFp2p	${\mathrm{rmsFPSNR}}_{1}\;p2p$	${\mathrm{rmsFPSNR}}_{2}\;p2p$	rmsFp2pl	${\mathrm{rmsFPSNR}}_{1}\;p2\mathrm{pl}$	${\mathrm{rmsFPSNR}}_{2}\;p2\mathrm{pl}$	Average Input Points	Average Output Points	Output/Input·100%
Azimuthal	2.4840	61.0363	−0.8851	0.5858	67.3331	11.7085	795,010	351,469	44.2094
Conic	2.4327	61.1258	−0.7061	0.6254	67.0423	11.1270	795,010	334,183	42.0351
Cylindrical	0.8799	65.5575	8.1574	0.2266	71.4296	19.9015	795,010	441,458	55.5286
Equalareacylindrical	1.8918	62.2127	1.4677	0.4537	68.4265	13.8954	795,010	361,188	45.4319
Equirectangular	0.9269	65.3283	7.6989	0.2354	71.2633	19.5689	795,010	428,221	53.8636
Mercator	0.8436	65.7380	8.5184	0.2199	71.5590	20.1603	795,010	449,793	56.5770
Miller 10-bit	**0.7681**	**66.1541**	**9.3506**	**0.1907**	**72.1825**	**21.4073**	795,010	449,260	56.5100
Miller 9-bit	0.8462	65.7263	8.4950	0.2203	71.5502	20.1427	795,010	449,260	56.5100
Pannini	0.9405	65.2639	7.5701	0.2402	71.1749	19.3922	795,010	410,965	51.6931
Rectilinear	1.0556	64.7615	6.5653	0.2653	70.7451	18.5326	795,010	374,173	47.0652
Stereographic	1.8777	62.2770	1.5964	0.4012	69.0208	15.0840	795,010	386,019	48.5552

**Table 7:** Point-to-point (p2p) and point-to-plane (p2pl) objective measures rmsF, ${\mathrm{rmsFPSNR}}_{1}$ and ${\mathrm{rmsFPSNR}}_{2}$, after Poisson reconstruction, using different projections with projection area of 1056 × 944 pixels (approximately 1,000,000), best values are bold; average input and output number of points and their ratio.

	rmsFp2p	${\mathrm{rmsFPSNR}}_{1}\;p2p$	${\mathrm{rmsFPSNR}}_{2}\;p2p$	rmsFp2pl	${\mathrm{rmsFPSNR}}_{1}\;p2\mathrm{pl}$	${\mathrm{rmsFPSNR}}_{2}\;p2\mathrm{pl}$	Average Input Points	Average Output Points	Output/Input·100%
Azimuthal	3.9483	59.2665	−4.4246	3.7950	59.4562	−4.0451	795,010	1,153,609	145.1062
Conic	3.6778	60.0292	−2.8992	3.4638	60.3884	−2.1809	795,010	1,153,016	145.0316
Cylindrical	1.8932	63.1058	3.2539	1.7200	64.0822	5.2069	795,010	1,155,024	145.2842
Equalareacylindrical	3.6355	59.5268	−3.9040	3.5119	59.6839	−3.5898	795,010	1,153,801	145.1304
Equirectangular	1.7756	63.3958	3.8340	1.6544	64.0645	5.1714	795,010	1,154,894	145.2679
Mercator	**1.6122**	**63.7848**	**4.6119**	**1.4535**	**64.7498**	**6.5421**	795,010	1,154,705	145.2441
Miller 10-bit	1.7871	63.3660	3.7744	1.6477	64.1868	5.4161	795,010	1,156,485	145.4680
Miller 9-bit	1.8834	63.1429	3.3282	1.7325	64.0029	5.0481	795,010	1,155,006	145.2819
Pannini	2.1112	62.6956	2.4336	1.9620	63.4910	4.0243	795,010	1,155,073	145.2904
Rectilinear	2.0828	62.6544	2.3512	1.9141	63.4887	4.0197	795,010	1,154,906	145.2694
Stereographic	3.5426	59.6347	−3.6883	3.4265	59.7821	−3.3934	795,010	1,155,261	145.3140

**Table 8:** Point-to-point (p2p) and point-to-plane (p2pl) objective measures rmsF, ${\mathrm{rmsFPSNR}}_{1}$ and ${\mathrm{rmsFPSNR}}_{2}$, after Poisson reconstruction, using different projections with projection area of 1296 × 1160 pixels (approximately 1,500,000), best values are bold; average input and output number of points and their ratio.

	rmsFp2p	${\mathrm{rmsFPSNR}}_{1}\;p2p$	${\mathrm{rmsFPSNR}}_{2}\;p2p$	rmsFp2pl	${\mathrm{rmsFPSNR}}_{1}\;p2\mathrm{pl}$	${\mathrm{rmsFPSNR}}_{2}\;p2\mathrm{pl}$	Average Input Points	Average Output Points	Output/Input·100%
Azimuthal	3.4226	59.8245	−3.3087	3.2923	60.0029	−2.9519	795,010	1,153,402	145.0802
Conic	3.0916	60.6366	−1.6844	2.9537	60.8878	−1.1820	795,010	1,152,742	144.9972
Cylindrical	1.3590	65.7201	8.4825	1.2248	67.0806	11.2035	795,010	1,154,542	145.2236
Equalareacylindrical	3.3320	60.1398	−2.6780	3.2203	60.3042	−2.3493	795,010	1,153,681	145.1153
Equirectangular	0.9471	66.7002	10.4428	0.8230	67.9619	12.9661	795,010	1,154,483	145.2162
Mercator	**0.6504**	**68.1203**	**13.2829**	**0.5124**	**69.8651**	**16.7726**	795,010	1,154,466	145.2140
Miller 10-bit	0.8328	67.7035	12.4494	0.7035	69.2895	15.6213	795,010	1,156,160	145.4271
Miller 9-bit	0.9335	67.2952	11.6327	0.8016	68.8654	14.7731	795,010	1,154,383	145.2036
Pannini	1.1857	65.9413	8.9250	1.0427	67.3226	11.6876	795,010	1,154,553	145.2250
Rectilinear	1.3954	64.9695	6.9813	1.2327	66.2226	9.4875	795,010	1,154,607	145.2318
Stereographic	2.4495	61.1509	−0.6558	2.3639	61.3091	−0.3394	795,010	1,155,021	145.2838

**Table 9:** Point-to-point (p2p) and point-to-plane (p2pl) objective measures rmsF, ${\mathrm{rmsFPSNR}}_{1}$ and ${\mathrm{rmsFPSNR}}_{2}$, after Poisson reconstruction, using different projections with projection area of 1496 × 1344 pixels (approximately 2,000,000), best values are bold; average input and output number of points and their ratio.

	rmsFp2p	${\mathrm{rmsFPSNR}}_{1}\;p2p$	${\mathrm{rmsFPSNR}}_{2}\;p2p$	rmsFp2pl	${\mathrm{rmsFPSNR}}_{1}\;p2\mathrm{pl}$	${\mathrm{rmsFPSNR}}_{2}\;p2\mathrm{pl}$	Average Input Points	Average Output Points	Output/Input·100%
Azimuthal	3.5851	59.9790	−2.9997	3.4530	60.1616	−2.6345	795,010	1,153,094	145.0414
Conic	4.0596	59.8137	−3.3304	3.9061	60.0175	−2.9226	795,010	1,152,734	144.9962
Cylindrical	**0.3314**	**69.8545**	**16.7514**	**0.2215**	**71.6639**	**20.3702**	795,010	1,154,352	145.1997
Equalareacylindrical	3.1122	60.9044	−1.1489	3.0077	61.0880	−0.7816	795,010	1,153,599	145.1050
Equirectangular	0.9305	67.6623	12.3669	0.8296	69.0639	15.1701	795,010	1,154,520	145.2208
Mercator	0.7053	68.7747	14.5918	0.6102	70.3353	17.7129	795,010	1,154,439	145.2106
Miller 10-bit	0.4555	69.8594	16.7611	0.3619	71.5899	20.2221	795,010	1,156,008	145.4080
Miller 9-bit	0.4522	69.8166	16.6757	0.3492	71.6585	20.3594	795,010	1,154,324	145.1962
Pannini	0.5384	68.7913	14.6249	0.4139	70.5697	18.1817	795,010	1,154,358	145.2004
Rectilinear	1.2472	66.2188	9.4800	1.1264	67.5502	12.1428	795,010	1,154,410	145.2070
Stereographic	2.8045	61.1347	−0.6883	2.6960	61.3675	−0.2226	795,010	1,155,019	145.2836

**Table 10:** Timing performance, for the *Longdress* point cloud and Miller 9-bit projection with panorama size of 2,000,000 points.

Timing Performance	Seconds per Point Cloud	Seconds per 20 Point Clouds
Point cloud to panorama:	1.9030	38.0603
Compression (texture):	-	117.3344
Decompression (texture):	-	2.0918
Compression (geometry):	-	0.9477
Decompression (geometry):	-	1.2366
Panorama to point cloud:	4.9350	98.6991
Normal calculation (CloudCompare):	6.2539	125.0783
Poisson reconstruction and upsampling (MeshLab):	97.0496	1941.0

**Table 11:** Octree reduction using 3DTK toolkit, with voxel size “R” = 1 and different parameters for randomized octree based point reduction with “O” points per voxel, for the *Longdress* point cloud.

*Longdress*, R = 1	O = 1	O = 2	O = 3	O = 4	O = 5	O = 6	O = 7	O = 8
Average number of output points:	230,014	427,083	575,585	698,867	761,164	793,732	794,969	795,009
Average size of .oct file, bytes:	3,684,335	6,837,436	9,213,473	11,185,991	12,182,742	12,703,824	12,723,616	12,724,261
Average number of input points:	795,010	795,010	795,010	795,010	795,010	795,010	795,010	795,010
Average bits per input point:	37.0746	68.8035	92.7130	112.5620	122.5921	127.8356	128.0348	128.0413
rmsFp2p:	0.7755	0.4700	0.2766	0.1209	0.0426	0.0016	0.0001	0.0000
${\mathrm{rmsFPSNR}}_{1}\;p2p$:	66.0727	68.2475	70.5507	74.1436	78.6783	92.9780	108.5374	Inf
${\mathrm{rmsFPSNR}}_{2}\;p2p$:	9.1877	13.5374	18.1437	25.3295	34.3989	62.9984	94.1172	Inf
rmsFp2pl:	0.2547	0.1700	0.1012	0.0499	0.0178	0.0007	0.0000	0.0000
${\mathrm{rmsFPSNR}}_{1}\;p2\mathrm{pl}$:	70.9081	72.6655	74.9173	77.9851	82.4677	96.4049	112.7170	Inf
${\mathrm{rmsFPSNR}}_{2}\;p2\mathrm{pl}$:	18.8587	22.3734	26.8770	33.0126	41.9778	69.8522	102.4764	Inf

**Table 12:** Octree reduction using 3DTK toolkit, with voxel size “R” = 2 and different parameters for randomized octree based point reduction with “O” points per voxel, for the *Longdress* point cloud.

*Longdress*, R = 2	O = 1	O = 2	O = 3	O = 4	O = 5	O = 6	O = 7	O = 8
Average number of output points:	60,161	116,599	170,613	222,186	270,673	317,488	362,572	406,039
Average size of .oct file, bytes:	966,646	1,869,673	2,733,914	3,559,090	4,334,882	5,083,923	5,805,268	6,500,740
Average number of input points:	795,010	795,010	795,010	795,010	795,010	795,010	795,010	795,010
Average bits per input point:	9.7271	18.8141	27.5107	35.8143	43.6209	51.1583	58.4171	65.4154
rmsFp2p:	1.5428	1.1679	0.9701	0.8358	0.7339	0.6492	0.5757	0.5099
${\mathrm{rmsFPSNR}}_{1}\;p2p$:	63.0855	64.2946	65.1006	65.7477	66.3126	66.8453	67.3669	67.8937
${\mathrm{rmsFPSNR}}_{2}\;p2p$:	3.2134	5.6316	7.2437	8.5378	9.6676	10.7330	11.7762	12.8298
rmsFp2pl:	0.3484	0.3121	0.2846	0.2599	0.2369	0.2152	0.1945	0.1748
${\mathrm{rmsFPSNR}}_{1}\;p2\mathrm{pl}$:	69.5486	70.0257	70.4259	70.8213	71.2238	71.6410	72.0803	72.5437
${\mathrm{rmsFPSNR}}_{2}\;p2\mathrm{pl}$:	16.1395	17.0939	17.8941	18.6850	19.4899	20.3244	21.2030	22.1299

**Table 13:** Octree reduction using 3DTK toolkit, with voxel size “R” = 1 and different parameters for randomized octree based point reduction with “O” points per voxel, for the *Redandblack* point cloud.

*Redandblack*, R = 1	O = 1	O = 2	O = 3	O = 4	O = 5	O = 6	O = 7	O = 8
Average number of output points:	211,582	389,317	517,726	620,888	672,157	699,481	701,144	701,234
Average size of .oct file, bytes:	3,389,106	6,232,878	8,287,429	9,938,021	10,758,325	11,195,502	11,222,110	11,223,546
Average number of input points:	795,010	795,010	795,010	795,010	795,010	795,010	795,010	795,010
Average bits per input point:	34.1038	62.7200	83.3945	100.0040	108.2585	112.6577	112.9255	112.9399
Symmetric rmsF p2p:	0.7591	0.4515	0.2622	0.1146	0.0415	0.0025	0.0001	0.0000
Symmetric PSNR_1 p2p:	66.1656	68.4226	70.7825	74.3788	78.7935	90.9982	103.9500	Inf
Symmetric PSNR_2 p2p:	10.4646	14.9786	19.6983	26.8910	35.7204	60.1298	86.0333	Inf
Symmetric rmsF p2pl:	0.2497	0.1630	0.0955	0.0472	0.0173	0.0011	0.0000	0.0000
Symmetric PSNR_1 p2pl:	70.9956	72.8476	75.1677	78.2340	82.5916	94.4710	108.0630	Inf
Symmetric PSNR_2 p2pl:	20.1246	23.8286	28.4687	34.6014	43.3166	67.0754	94.2595	Inf

**Table 14:** Octree reduction using 3DTK toolkit, with voxel size “R” = 2 and different parameters for randomized octree based point reduction with “O” points per voxel, for the *Redandblack* point cloud.

*Redandblack*, R = 2	O = 1	O = 2	O = 3	O = 4	O = 5	O = 6	O = 7	O = 8
Average number of output points:	55,189	106,830	156,077	202,877	246,949	289,386	330,106	369,273
Average size of .oct file, bytes:	886,801	1,713,077	2,501,036	3,249,837	3,954,998	4,633,979	5,285,505	5,912,168
Average number of input points:	795,010	795,010	795,010	795,010	795,010	795,010	795,010	795,010
Average bits per input point:	8.9237	17.2383	25.1673	32.7023	39.7982	46.6306	53.1868	59.4928
Symmetric rmsF p2p:	1.5165	1.1475	0.9520	0.8189	0.7171	0.6323	0.5586	0.4924
Symmetric PSNR_1 p2p:	63.1605	64.3712	65.1824	65.8364	66.4132	66.9598	67.4984	68.0461
Symmetric PSNR_2 p2p:	4.4544	6.8758	8.4982	9.8061	10.9599	12.0530	13.1301	14.2255
Symmetric rmsF p2pl:	0.3515	0.3120	0.2826	0.2568	0.2330	0.2106	0.1894	0.1694
Symmetric PSNR_1 p2pl:	69.5091	70.0272	70.4576	70.8731	71.2956	71.7355	72.1946	72.6804
Symmetric PSNR_2 p2pl:	17.1515	18.1877	19.0486	19.8797	20.7245	21.6044	22.5225	23.4941

**Table 15:** Octree reduction using 3DTK toolkit, with voxel size “R” = 1 and different parameters for randomized octree based point reduction with “O” points per voxel, for the *Soldier* point cloud.

*Soldier*, R = 1	O = 1	O = 2	O = 3	O = 4	O = 5	O = 6	O = 7	O = 8
Average number of output points:	298,525	554,593	753,226	920,279	1,011,060	1,058,928	1,060,749	1,060,770
Average size of .oct file, bytes:	4,781,816	8,878,917	12,057,034	14,729,884	16,182,391	16,948,270	16,977,416	16,977,739
Average number of input points:	795,010	795,010	795,010	795,010	795,010	795,010	795,010	795,010
Average bits per input point:	48.1183	89.3465	121.3271	148.2234	162.8396	170.5465	170.8398	170.8430
Symmetric rmsF p2p:	0.7867	0.4851	0.2906	0.1324	0.0469	0.0017	0.0000	0.0000
Symmetric PSNR_1 p2p:	66.0108	68.1101	70.3362	73.7484	78.2606	92.5742	112.3807	Inf
Symmetric PSNR_2 p2p:	6.5947	10.7933	15.2455	22.0699	31.0944	59.7214	99.3344	Inf
Symmetric rmsF p2pl:	0.2672	0.1826	0.1110	0.0557	0.0199	0.0008	0.0000	0.0000
Symmetric PSNR_1 p2pl:	70.7010	72.3536	74.5152	77.5116	81.9802	95.8459	116.2297	Inf
Symmetric PSNR_2 p2pl:	15.9752	19.2803	23.6035	29.5964	38.5334	66.2649	107.0324	Inf

**Table 16:** Octree reduction using 3DTK toolkit, with voxel size “R” = 2 and different parameters for randomized octree based point reduction with “O” points per voxel, for the *Soldier* point cloud.

*Soldier*, R = 2	O = 1	O = 2	O = 3	O = 4	O = 5	O = 6	O = 7	O = 8
Average number of output points:	78,169	151,269	221,122	287,916	350,702	411,306	469,731	526,048
Average size of .oct file, bytes:	1,256,069	2,425,706	3,543,359	4,612,076	5,616,648	6,586,314	7,521,122	8,422,194
Average number of input points:	795,010	795,010	795,010	795,010	795,010	795,010	795,010	795,010
Average bits per input point:	12.6395	24.4093	35.6560	46.4102	56.5190	66.2765	75.6833	84.7506
Symmetric rmsF p2p:	1.5565	1.1815	0.9842	0.8503	0.7489	0.6649	0.5922	0.5272
Symmetric PSNR_1 p2p:	63.0473	64.2443	65.0378	65.6730	66.2245	66.7409	67.2443	67.7490
Symmetric PSNR_2 p2p:	0.6678	3.0617	4.6487	5.9190	7.0221	8.0549	9.0618	10.0711
Symmetric rmsF p2pl:	0.3544	0.3203	0.2942	0.2705	0.2483	0.2271	0.2067	0.1871
Symmetric PSNR_1 p2pl:	69.4744	69.9128	70.2828	70.6468	71.0190	71.4072	71.8164	72.2488
Symmetric PSNR_2 p2pl:	13.5218	14.3986	15.1387	15.8666	16.6112	17.3875	18.2058	19.0707

## Data Availability

The data presented in this study are openly available at http://msl.unin.hr/ accessed on 13 September 2021.
